# Genome-wide CRISPRi screens for high-throughput fitness quantification and identification of determinants for dalbavancin susceptibility in *Staphylococcus aureus*

**DOI:** 10.1128/msystems.01289-23

**Published:** 2024-06-05

**Authors:** Xue Liu, Vincent de Bakker, Maria Victoria Heggenhougen, Marita Torrissen Mårli, Anette Heidal Frøynes, Zhian Salehian, Davide Porcellato, Danae Morales Angeles, Jan-Willem Veening, Morten Kjos

**Affiliations:** 1 Department of Pathogen, Biology, International Cancer Center, Shenzhen University Medical School, Shenzhen, Guangdong, China; 2 Department of Fundamental Microbiology, University of Lausanne, , Switzerland; 3 Faculty of Chemistry, Biotechnology and Food Science, Norwegian University of Life Sciences, Norway; Teagasc Food Research Centre Moorepark, Fermoy, Cork, Ireland

**Keywords:** CRISPR interference, *Staphylococcus aureus*, genetic screen, antibiotic resistance

## Abstract

Antibiotic resistance and tolerance remain a major problem for the treatment of staphylococcal infections. Identifying genes that influence antibiotic susceptibility could open the door to novel antimicrobial strategies, including targets for new synergistic drug combinations. Here, we developed a genome-wide CRISPR interference library for *Staphylococcus aureus*, demonstrated its use by quantifying gene fitness in different strains through CRISPRi-seq, and used it to identify genes that modulate susceptibility to the lipoglycopeptide dalbavancin. By exposing the library to sublethal concentrations of dalbavancin using both CRISPRi-seq and direct selection methods, we not only found genes previously reported to be involved in antibiotic susceptibility but also identified genes thus far unknown to affect antibiotic tolerance. Importantly, some of these genes could not have been detected by more conventional transposon-based knockout approaches because they are essential for growth, stressing the complementary value of CRISPRi-based methods. Notably, knockdown of a gene encoding the uncharacterized protein KapB specifically sensitizes the cells to dalbavancin, but not to other antibiotics of the same class, whereas knockdown of the Shikimate pathway showed the opposite effect. The results presented here demonstrate the promise of CRISPRi-seq screens to identify genes and pathways involved in antibiotic susceptibility and pave the way to explore alternative antimicrobial treatments through these insights.

**IMPORTANCE:**

Antibiotic resistance is a challenge for treating staphylococcal infections. Identifying genes that affect how antibiotics work could help create new treatments. In our study, we made a CRISPR interference library for *Staphylococcus aureus* and used this to find which genes are critical for growth and also mapped genes that are important for antibiotic sensitivity, focusing on the lipoglycopeptide antibiotic dalbavancin. With this method, we identified genes that altered the sensitivity to dalbavancin upon knockdown, including genes involved in different cellular functions. CRISPRi-seq offers a means to uncover untapped antibiotic targets, including those that conventional screens would disregard due to their essentiality. This paves the way for the discovery of new ways to fight infections.

## INTRODUCTION

The spread of antibiotic resistance in pathogenic bacteria combined with a halt in the antibiotic drug development pipeline represents a major threat to global health. Resistant *Staphylococcus aureus*, and particularly methicillin-resistant *S. aureus* (MRSA), is among the drug-resistant pathogens responsible for most deaths worldwide (1). For the treatment of infections caused by resistant Gram-positive bacteria, glycopeptide antibiotics such as vancomycin or vancomycin derivatives and the lipopeptide daptomycin are commonly used (2, 3). However, although they are potent antimicrobial agents, resistance to these antibiotics is also rising (4). A continued effort to decipher mechanisms of resistance and identify novel drugs and combinatorial therapies as well as new drug targets is therefore critical. High-throughput genetics techniques linking genotypes to antibiotic susceptibility represent an attractive approach to identify potential novel combination therapies to combat resistance or resensitize antibiotic-resistant bacteria. For example, transposon insertion sequencing (Tn-seq) has been used to identify genetic determinants involved in susceptibility to daptomycin in *S. aureus* (5). One major limitation to such Tn-seq-based analyses, however, is that essential targets will not be included in the analyses as such mutants will not survive during library construction.

An approach to circumvent this is to perform genome-wide depletion of gene expression using CRISPR interference (CRISPRi). With CRISPRi, a catalytically inactive Cas9, known as dCas9, which can bind but not cleave DNA, is harnessed for repression of transcription (6, 7). dCas9 is co-expressed with a single-guide RNA (sgRNA), a gene-specific RNA molecule that guides the dCas9 protein to its target sequence. The sgRNA consists of a 20-nt long target-specific sequence and a so-called Cas9 handle that is important for interaction with dCas9. By replacing the gene-specific sequence, the CRISPRi system can be directed to a new target. When bound within a gene, the dCas9-sgRNA complex acts as a transcriptional roadblock to block RNA polymerase, thereby inhibiting transcription (6, 7). Thus, CRISPRi will also inhibit the expression of other genes co-transcribed with the target gene. CRISPRi has proven highly useful for functional studies of essential genes in a diversity of bacteria, including *S. aureus* (8–12). The relative simplicity and programmability of CRISPRi have also allowed upscaling to genome-wide CRISPRi libraries in bacterial species such as *Escherichia coli* (13–15), *Streptococcus pneumoniae* (16), *Streptococcus salivarius* (17), *Bacillus subtilis* (18), *Mycobacterium tuberculosis* (19), *Acinetobacter baumanii* (20), and *Vibrio natriegens* (21). Using a CRISPR adaptation strategy to generate guide RNAs, a CRISPRi library with an average of 100 sgRNAs targeting each gene has also been developed for *S. aureus* (22). With such pooled CRISPRi libraries, the fitness of every single operon can be determined under different growth conditions by quantifying sgRNAs in the libraries with Illumina sequencing (termed CRISPRi-seq) (16, 23). CRISPRi-seq can be used to identify new targets for antibiotic therapy, and we recently used CRISPRi-seq to show that amoxicillin-resistant *S. pneumoniae* can be resensitized to this antibiotic by combining it with the U.S. Food and Drug Administration (FDA)-approved fertility drug clomiphene (24). In this work, we designed and developed a compact, genome-wide-inducible CRISPR interference library for *S. aureus* strain NCTC8325 and used the library to quantify gene fitness and screen for genes influencing the susceptibility to the clinically relevant lipoglycopeptide antibiotic dalbavancin.

Dalbavancin is a semi-synthetic lipoglycopeptide with broad activity against Gram-positive bacteria including staphylococci, streptococci, and enterococci (25). Like other glycopeptide antibiotics such as vancomycin and teicoplanin, dalbavancin targets cell wall synthesis by binding to the D-ala-D-ala dipeptide terminus of the lipid II, thereby sequestering this molecule, inhibiting peptidoglycan polymerization and crosslinking. Dalbavancin has a typical heptapeptide core that is modified compared with other glycopeptides and carries a lipid side chain. This side chain may be involved in its mechanism of action by anchoring the molecule to the bacterial cell membrane (26) and/or interacting with charged phospholipid head groups (27), although this has not been established. Dalbavancin is a highly potent anti-MRSA drug and has also been shown to be active against strains with reduced susceptibility to vancomycin or the last-resort lipopeptide drug daptomycin (28). The drug has been approved for use against acute bacterial skin and skin-structure infections (ABSSSIs) (29) but is also used for the treatment of endocarditis and osteomyelitis (30). Dalbavancin has unique pharmacokinetic properties, with a half-life of more than a week, allowing for once-weekly administration of the drug (31, 32).

Although still rare, dalbavancin non-susceptible mutants have been isolated from patients (33). One of these was shown to harbor mutations in *yvqF* (*vraT*), a membrane protein encoded in the same operon as the two-component system VraSR. VraSR is known to regulate cell wall metabolism (34) and has previously been associated with vancomycin-intermediate *S. aureus* (VISA) strains (35). *In vitro* exposure of *S. aureus* to dalbavancin has also been shown to result in selection of tolerant mutants harboring mutations in genes associated with VISA strains (35, 36). Although vancomycin-resistant *S. aureus* (VRSA) strains are resistant to high glycopeptide concentrations due to the *vanA* resistance operon (2) causing direct target modification, VISA strains have a moderate reduction in susceptibility to glycopeptides. VISA is more prevalent than VRSA and represents a major problem for the treatment of infections (35), and it is therefore critical to get better insights into the mechanism of action and understand which factors affect susceptibility in such cases. VISA strains mostly carry mutations that alter the cell surface to hinder the antibiotic from reaching its target and are often characterized by a thickened cell wall and altered transpeptidation cross-linking activity (35).

Here, we constructed a genome-wide CRISPRi library in the commonly used laboratory strain *S. aureus* NCTC8325-4, a prophage-rescued derivative of NCTC8325, and performed CRISPRi-seq screens to quantify genome-wide gene fitness and identify genes modulating the susceptibility to dalbavancin in *S. aureus*.

## RESULTS

### Adapting the IPTG-inducible CRISPRi system in *S. aureus* to facilitate library construction

**Fig 1:**
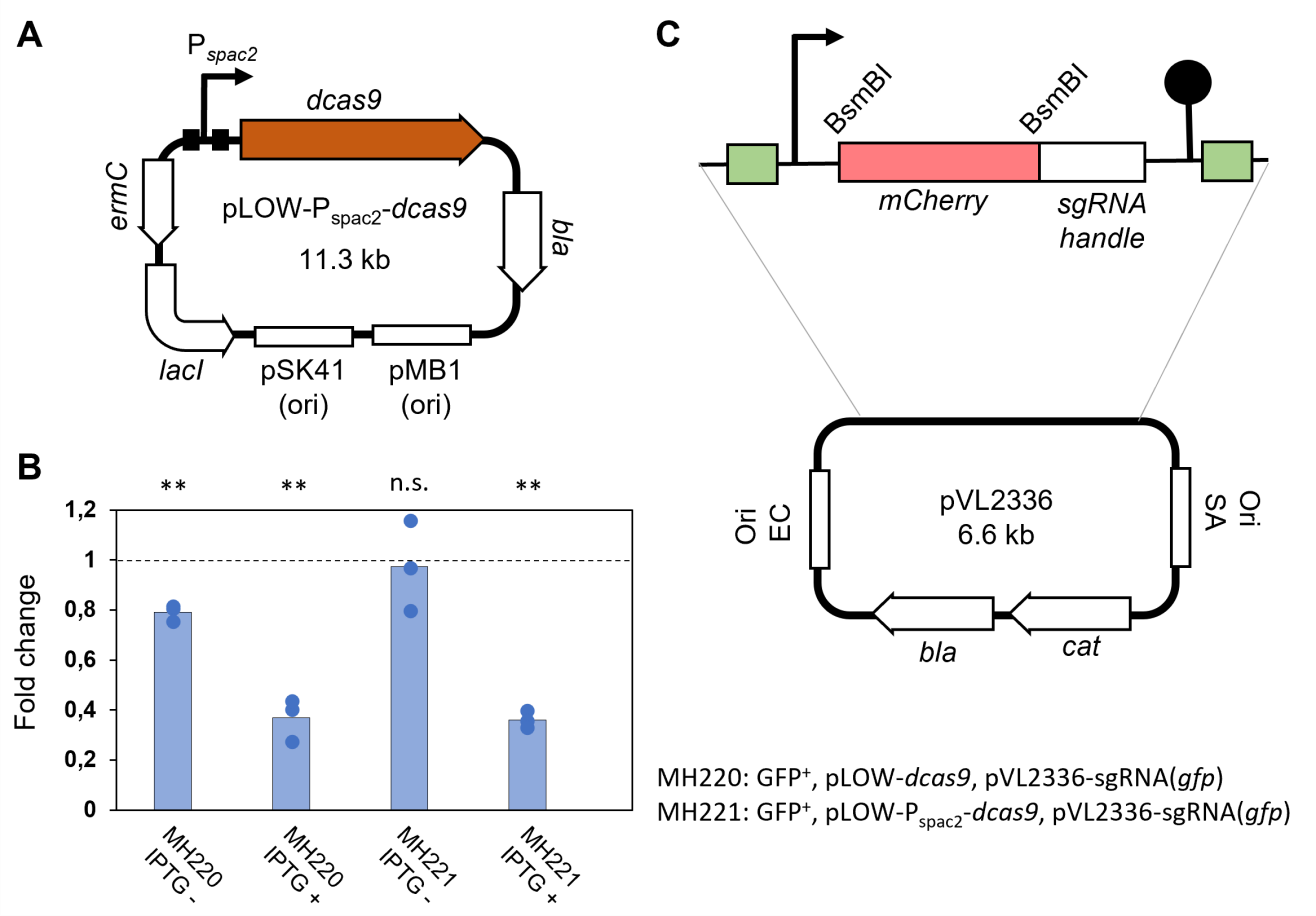
Adaptation of the staphylococcal CRISPRi system for CRISPRi-seq. (**A)**) Schematic representation of pLOW-P*_spac2_-dcas9*. The two *lacO* sites are indicated by black boxes. (**B)**) Reporter assay comparing background repression of GFP expression in CRISPRi-strains with the original system pLOW-dcas9 (in strain MH220) and the novel pLOW-P*_spac2_-dcas9* (in strain MH221). Exponentially growing cells were induced with 250 µM IPTG, and relative fluorescence units (RFU)/optical density (OD) were measured after 90-min depletion. Average fold change in RFU/OD relative to a control strain constitutively expressing GFP (strain MK1482, indicated by the dashed line) is plotted, along with data points from the three individual replicates. Two-sample *t*-tests, comparing each of the test strains with the control, were performed, and significance is indicated by asterisks (*P* < 0.001), n.s.; not significantly different. (**C)**) Schematic representation of the pVL2336-plasmid. The green boxes represent the flanking Illumina amplicon sequences.

In our previously described two-plasmid, isopropyl β-D-1-thiogalactopyranoside (IPTG)-inducible CRISPRi system (12), *dcas9*-expression was controlled by a P_spac_ promoter containing a single *lacO* operator site in the plasmid pLOW-dcas9, whereas the sgRNA was constitutively expressed in plasmid pCG248-sgRNA(xxx) (12, 37). To reduce the background expression of *dcas9* in uninduced conditions, a second *lacO*-operator was inserted into the promoter to generate plasmid pLOW-P_spac2_-*dcas9* (Fig. 1A). To compare the dynamic range of induction for the two promoters, an *S. aureus* strain (MK1482) constitutively expressing GFP from the chromosome was used as a reporter. The *dcas9*-plasmids, as well as pCG248-sgRNA(*gfp*), expressing a *gfp*-targeting sgRNA, were transformed into the *S. aureus* GFP reporter strain, and GFP expression was measured with and without IPTG induction (90-min induction) (Fig. 1B). pLOW-P_spac2_-*dcas9* indeed showed a tighter regulation of *dcas9* compared with the strains carrying the original plasmid pLOW-*dcas9* in this reporter assay, and the former plasmid was therefore chosen for further use.

In our original CRISPRi system, we used inverse PCR to insert sgRNAs into plasmid pCG248-sgRNA(xxx) (12). To aid library construction, a new sgRNA vector (pVL2336) was engineered to allow insertion of sgRNAs by Golden Gate cloning using the class IIs restriction enzyme BsmBI (Fig. 1C). In pVL2336, two BsmBI restriction sites were inserted to flank an *mCherry* reporter gene placed exactly at the position of the 20-nt sgRNA base pairing region, thus allowing for red-white screening during Golden Gate cloning of new sgRNAs. In order to replace *mCherry* with 20-nt sgRNA base-pairing regions, a forward and a reverse oligo, each of 24 nt (20-nt base pairing region and 4-nt overhangs to the digested plasmid), were designed, and the two oligos were annealed and ligated into digested pVL2336, using a similar strategy as before (16). Furthermore, to enable one-step PCR during library preparation for amplicon sequencing, read 1, read 2, and adaptor sequences for Illumina sequencing were inserted in the sgRNA flanking sites.

### A genome-wide *S. aureus* CRISPRi library with 1,928 sgRNAs provides wide coverage of multiple strains

The genomic features and transcriptional units of *S. aureus* NCTC8325 defined by Mäder et al. (38) were used as a starting point for the design of sgRNA target sequences for a genome-wide *S. aureus* CRISPRi library. In total, 4,028 genomic features have been annotated in the *S. aureus* NCTC8325 genome (accession number NC_007795/GCF_00013425), including open reading frames (ORFs), small RNAs, and tRNAs. Genome-wide gene targeting in *S. aureus* is mainly restricted by the low transformation efficiency, which makes large sgRNA pools very challenging to construct. To get a concise, efficient, and balanced CRISPRi library, we decided to only include the ORFs and tRNAs in the library, resulting in 2,836 features. The 1,000 small RNAs were thus not directly covered in this study. Among the targeted features, 1,041 are ORFs in transcription units (TUs) encoded on the positive strand; 1,159 features are ORFs in TUs on the negative strand, and 636 features are not ascribed to any TU (38).

**Fig 2:**
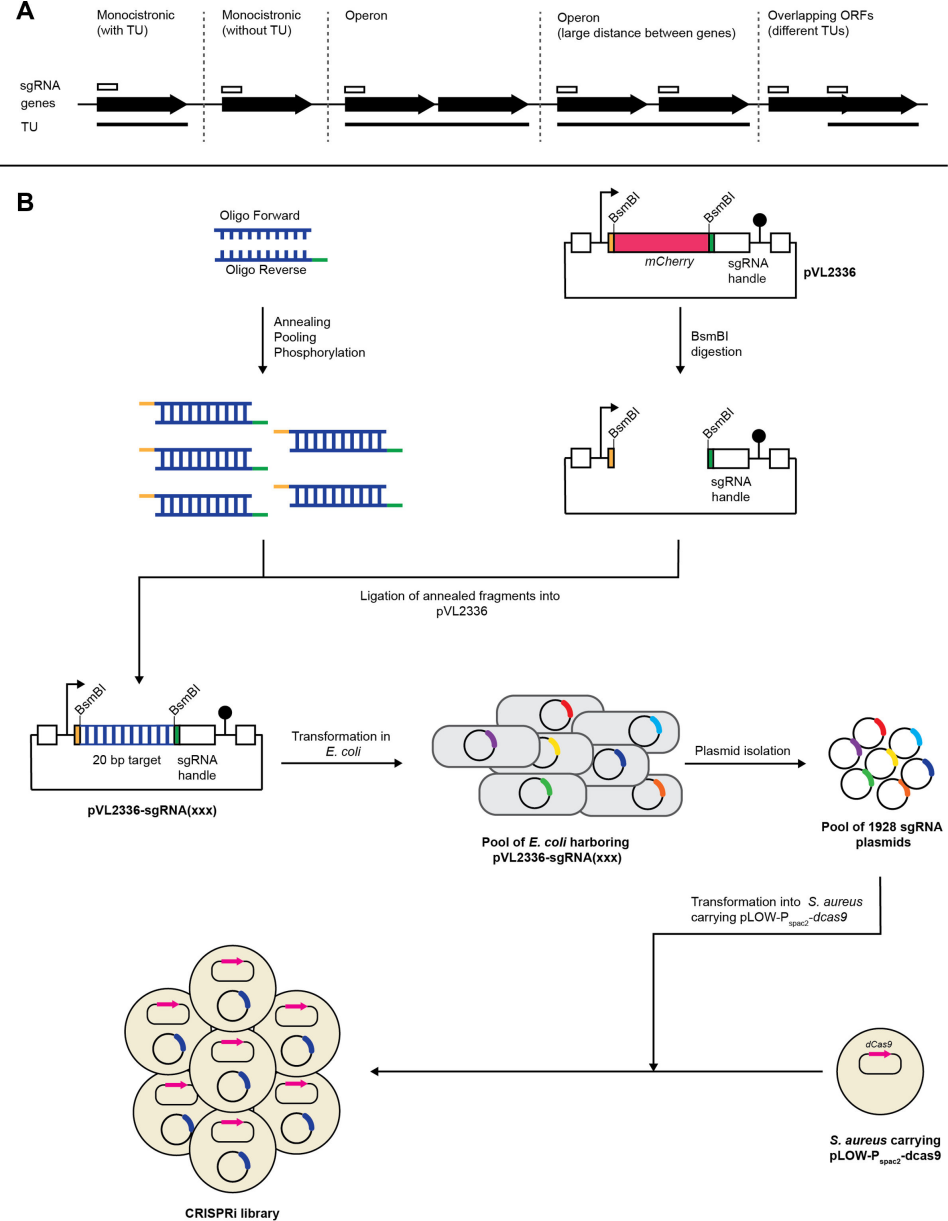
The *S. aureus* CRISPRi library. (**A**) Design strategy for sgRNAs in the CRISPRi library. Genes are indicated as arrows, TUs as black lines, and sgRNAs as white boxes. (**B**) Schematic outline of the construction of the CRISPRi library. For each sgRNA, two 24-nt oligos were designed, with 4-nt overhang on each end to be compatible with the digested vector. The two oligos were annealed, and then, the 1,928 annealing products were pooled into one tube as the insert fragments for the following Golden Gate cloning. The plasmid pVL2336 was digested with BsmBI, and then, the backbone was purified. The annealing pool was then ligated into digested pVL2336, followed by transformation into *E. coli* IM08B. The transformants were collected and pooled together as a reservoir for sgRNA plasmids. The sgRNA plasmids were then transformed into *S. aureus* with IPTG-inducible *dcas9*.

The following strategy was used for sgRNA design to cover all transcriptional units of the NCTC8325-4 genome (Fig. 2A): (i) an sgRNA was designed for all monocistronic ORFs; (ii) for the TUs with several ORFs, only the first ORF was selected as sgRNA target, since the same sgRNA will repress downstream genes due to polar effects of the CRISPRi approach; (iii) in addition, to reduce the risk of losing any genes in the library due to mis-annotation of the TUs, we introduced an extra sgRNA when the distance between two ORFs inside a predicted TU was more than 100 bp; and (iv) for the 636 ORFs not ascribed to any TU, one sgRNA was designed for each of the individual ORFs (Fig. 2A).

sgRNAs were designed to target the 5’ end of the selected genes (see Materials and Methods for details). In total, 1,928 sgRNAs (Table S1) were designed to cover 2,764 out of the total 2,836 features (Table S2) in the NC_007795 genome used for sgRNA design (thus covering >97% of all genomic features). The remaining 72 features in the NCTC8325 genome were not included in the library due to the lack of protospacer adjacent motif (PAM) sequences. Detailed information about the CRISPRi library, including a list of all sgRNA sequences (Table S1), an overview of all NCTC8325 locus tags along with corresponding sgRNA (Table S2), and a list of the non-targeted locus tags (Table S3) can be found in the supplemental material.

Our manually designed sgRNA library for NCTC8325 was evaluated for specificity and potential off-target effects according to de Bakker et al. (23). Eight sgRNAs were found to have more than one exact binding site in the genome, of which two not on the template strand within an annotated gene (Table S1). Specifically, sgRNA0034, sgRNA0179, sgRNA0929, sgRNA1456, sgRNA1900, and sgRNA1901 target conserved regions among gene sets SAOUHSC_00052–4, SAOUHSC_00269/00275/00276, SAOUHSC_01511/01512/01584, SAOUHSC_01410/01805/01881/02437, SAOUHSC_A01910/A01909, and SAOUHSC_A02013/00786, respectively. The former two sets are considered potential paralogs according to KEGG Sequence Similarity Database (SSDB) (39). The off-target analysis further revealed that some of these and a few other sgRNAs (sgRNA0268, sgRNA1294, and sgRNA1580) have a relatively high expected off-target repression activity (Table S1), again partly due to potential paralogs. Some caution is therefore warranted when interpreting enrichment effects for these sgRNAs.

We also used the same pipeline to evaluate the sgRNA library functionality and genome coverage in seven other, commonly used *S. aureus* strains: Newman, JE2 USA300, COL, DSM20231, Mu50, RF122, and MRSA252 (Tables S4 to S10). We estimate that the library covers 79% to 93% of all annotated features in those genomes, assuming similar operon structures and polar effects compared with the NCTC8325 strain, for which the library was designed for (Fig. S1A). Note that this only concerns exact binding sites, meaning potentially even higher coverages when also considering those features with minor allelic differences, causing imperfect sgRNA binding in some strains. This indicates that the library could potentially be used across different *S. aureus* strains.

To construct the CRISPRi library, we first made a plasmid pool with 1,928 sgRNAs in *E. coli* IM08B. The 20 bp base-pairing region of the sgRNAs was first cloned into the above-mentioned *S. aureus–E. coli* shuttle vector, pVL2336, by pooled Golden Gate cloning and then transformed into competent *E. coli* IM08B (Fig. 2B). In total, 1.3 × 10^6^ transformant colonies were obtained, providing a theoretical 674-fold coverage of the 1,928-sgRNA library. The false-positive ratio of sgRNA cloning was estimated to be lower than 0.03% (no mCherry-positive colonies were identified among 3,000 colonies). All the transformant colonies were pooled into one tube as a reservoir of sgRNA plasmids to construct the CRISPRi libraries in different *S. aureus* strains. To test the coverage and distribution of the 1,928 sgRNAs, plasmids were purified from the pooled *E. coli* IM08B transformants and used as template in the PCR reaction for Illumina library preparation. Only one sgRNA (targeting locus tag SAOUHSC_01984) was missing from the pool, confirming that the sgRNA plasmid pool was well-constructed, with sufficient coverage. Consistently, the distribution of the sgRNA counts confirmed that the library was well-balanced, as shown in Fig. S2. The plasmid pool was then transformed into *S. aureus* NCTC8325-4 carrying pLOW-P_spac2_-*dcas9* (Fig. 2B) in two independent experiments (library 1 and library 2). For each experiment, >200,000 colonies were collected and pooled for the final construction of the *S. aureus* CRISPRi library.

### CRISPRi-seq for genome-wide fitness quantifications in *S. aureus*

**Fig 3:**
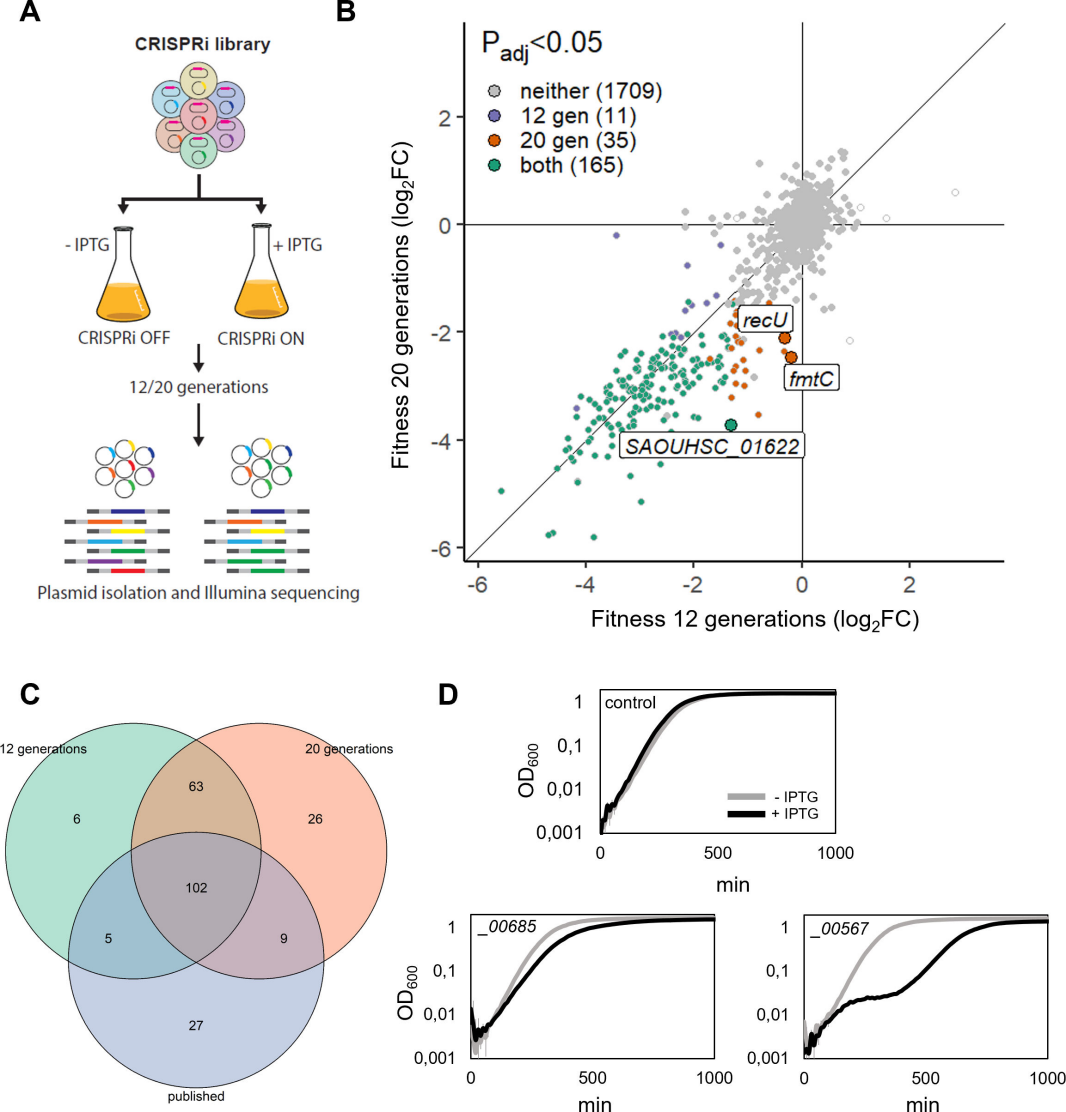
Gene fitness analysis of NCTC8325-4 by CRISPRi-seq. (**A**) Flowchart of the CRISPRi-seq screen. (**B**) Fitness effects upon *dcas9* induction for 12 versus 20 generations of exponential growth. Genes with significant differential fitness effects (|Δlog_2_FC| > 1, *P*_adj_ < 0.05) are depicted larger and alongside sgRNA target names. Points without color represent sgRNAs for which no *P*-value was determined due to extreme count outliers as detected by DESeq2. (**C**) Venn diagram showing the comparison of genes with significant fitness defect by CRISPRi-seq and essentialomes as defined by Tn-seq-based screens of related strains. (**D**) Experimental verification showing knockdown of two genes in NCTC8325-4 not defined as essential in previous screens. Identifiers correspond to SAOUHSC_ locus tags; 500 µM IPTG was added for induction. The control strain harbors a non-targeting sgRNA (strain MM75). Averages of triplicates with standard errors are shown.

To test the potential of the constructed CRISPRi library, we performed CRISPRi-seq to quantify the fitness of the individual sgRNA targets in this strain. The library was grown in brain heart infusion (BHI) medium in the presence or absence of IPTG for 12 (library 1) or 20 generations (library 2), plasmids were isolated, and the sgRNA abundances were determined by Illumina sequencing (Fig. 3A; Table S11). Most of the CRISPRi strain heterogeneity between samples could indeed be attributed to *dcas9* induction (Fig. S3). We computed a fitness score for each sgRNA based on the change in abundance after induction, and changes were defined as essential if log_2_FC < −1 and *P*_adj_ < 0.05.

We found 176 sgRNAs to have significantly reduced abundance after 12 generations of growth, and 200 after 20 generations (Fig. 3B; Table S12). A differential fitness analysis to directly compare the two conditions, showed that only three sgRNAs were found to be significantly different between the 12- and 20-generation experiments (|Δlog_2_FC| > 1, *P*_adj_ < 0.05) (Fig. 3B; Table S12), demonstrating that most of the genes with major effects on fitness are already evident after 12 generations of growth.

We compared *S. aureus* NCTC8325-4 target genes with significant fitness defects with essential genes as defined in previous Tn-seq studies in other *S. aureus* NCTC8325-derived strains, including *S. aureus* HG001 grown in tryptic soy broth (TSB) (40) or BHI (41) and *S. aureus* SH1000 in BHI (42). Across these Tn-seq analyses, 212 genes were found to be essential across strains and growth media. These 212 conserved essential genes were targeted by 143 sgRNAs in our library, and we found that out of those, 75% (107 sgRNAs) and 78% (111 sgRNAs) also showed significantly reduced abundance by CRISPRi-seq in the 12- and 20-generation experiments, respectively (Fig. 3C), indicating consistency between Tn-seq and CRISPRi-seq outcomes. Among the remaining sgRNAs expected to target essential genes, several have a relatively large, but non-significant reduction in sgRNA abundance upon induction (e.g., SAOUHSC_00009 and SAOUHSC_00225 in the 20-generation experiment).

There are 63 sgRNA targets that have significantly reduced abundance in both our CRISPRi-seq screens, although not being defined as conserved essential across the published transposon-based essentiality screens (Fig. 3C). Of these 63 targeted genes, 48 are defined as essential in at least one of the mentioned screens (40–42), suggesting that their essentiality is condition- or strain-dependent. Hence, 15 sgRNAs were significantly depleted in the CRISPRi-seq screen, but their target genes have not previously been defined as essential for growth in rich media (Table S13). We suspected that these might be explained by the well-documented polar effects of the CRISPRi system. As expected, nine sgRNAs most likely affect essential genes immediately up- or downstream of the target (Table S13). Of the six remaining sgRNAs, one targets *ccpA* (encoding the catabolite control protein CcpA, which governs metabolic regulation ), three target tRNAs, and two target genes of unknown function (SAOUHSC_00567 and SAOUHSC_00685). The growth defect resulting from knockdown of these last two genes was confirmed by individually constructed CRISPRi mutants (Fig. 3D). We note that we cannot exclude that dCas9-related polar effects play a role in the fitness defect shown for these genes, and this awaits future experimental validation.

Our sgRNA efficiency and specificity analysis showed that >89% of the sgRNAs should also be functional in *S. aureus* Newman (Fig. S1). To show the potential of the sgRNA pool for multiple *S. aureus* strains, we performed a similar CRISPRi-seq screen in *S. aureus* Newman. A 12-generation CRISPRi-seq experiment was performed in the same way as shown in Fig. 2A. From this experiment, 202 sgRNA targets were shown to have significantly reduced abundance in *S. aureus* Newman (log_2_FC<-1, *P*_adj_ < 0.05) (Fig. S4A; Tables S14 and S15). A total of 87% of the sgRNAs (153 sgRNAs of 175) with significantly reduced abundance in the NCTC8325-4 experiment after 12 generations was also found to have the same effect in Newman (Fig. S4). In contrast, while CRISPRi-seq with the NCTC8325-4 library did not identify any sgRNA target as costly [i.e., significantly increased abundance (log_2_FC > 1, *P*_adj_ < 0.05)], nine such targets were found in Newman, meaning that the depletion of these genes resulted in improved fitness within the pooled library experiment (Fig. S4). Interestingly, most of these targets (with the exception of the uncharacterized genes NWMN_0678 and NWMN_1673) have been associated with cell surface properties of staphylococcal cells, including biosynthesis or modification of teichoic acid (*dlt*-locus for sgRNA0545, *tagA*, and *fmtA*) (43–45), peptidoglycan (*oatA*) (46), or two-component systems regulating such properties (*saeRS*, *graXRS,* and associated *vraFG*-locus) (47, 48). We constructed single CRISPRi strains to knock down these seven targets (*dlt*-locus, *tagA*, *fmtA*, *oatA*, *saeRS*, *graXRS*, and *vraFG*); however, the growth of these single strains was not different from that of the control. Under growth in rich media, as was used here, the high growth rate of the control strain is probably difficult to improve for any mutant. This suggests that the expected fitness gain by knockdown of these genes will only occur during more complex competition settings, which is the case during growth of the pooled library experiment.

### CRISPRi-seq reveals factors important for susceptibility to the lipoglycopeptide dalbavancin

**Fig 4:**
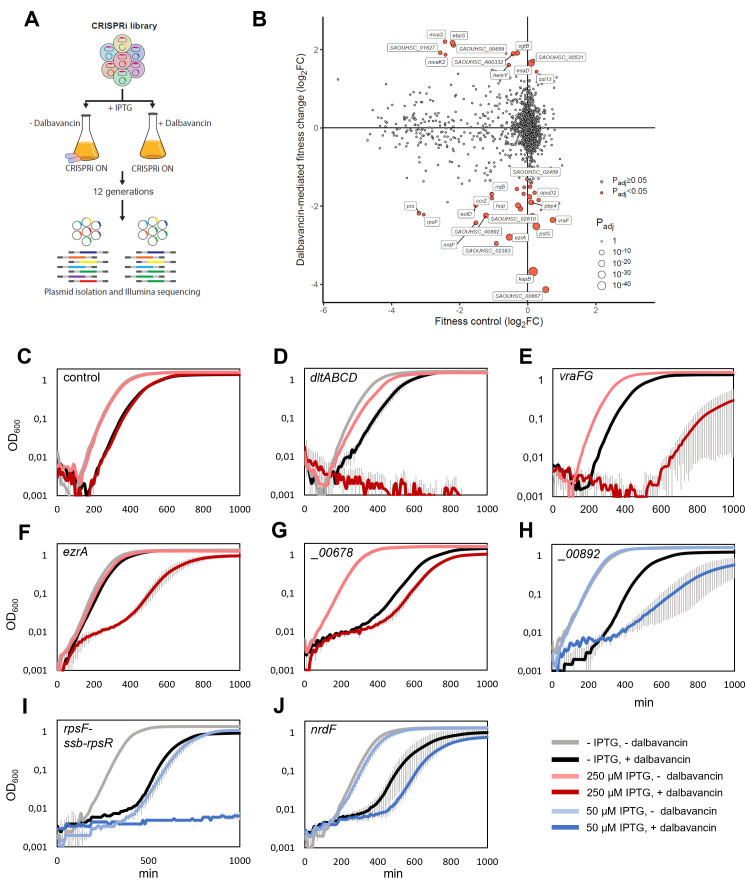
CRISPRi-seq identified factors affecting dalbavancin susceptibility. (**A**) Schematic workflow of the screen with dalbavancin stress. (**B**) CRISPRi-seq identified genes related to *S. aureus* sensitivity toward dalbavancin. Gene fitness was evaluated by CRISPRi-seq comparing CRISPRi-induced samples with a sublethal dose of dalbavancin to induced samples without dalbavancin (*y*-axis). This was contrasted with background fitness values as quantified with 12 generations of CRISPRi induction (*x*-axis, same as Fig. 3B). Targets of sgRNAs showing a significant change in fitness upon dalbavancin treatment were highlighted in red (|log_2_FC| > 1, *P*_adj_ < 0.05). The top hits selected for confirmation were labeled with their targeted genes. (**C–J**) Confirmation of the screen with individual CRISPRi mutants. The CRISPRi system was induced with either 250 µM IPTG (red) or 50 µM IPTG (blue) to reach an appropriate degree of knockdown. Cells were grown with or without sublethal concentrations of dalbavancin (0.015 µg/mL for panels C, D, and F or 0.03 µg/mL for panels E, G, H, I, and J). Non-targeting sgRNA-vector was used as control (**C**). Targeted genes were *dltA* (**D**), *vraFG* (**E**), *ezrA* (**F**), SAOUHSC_00678 (**G**), SAOUHSC_ 00892 (**H**), *rpsF-ssb-rpsR* (**I**), and *nrdF* (**J**). Averages of triplicates with standard errors are shown.

Dalbavancin is a lipoglycopeptide antibiotic whose primary mechanism of action is to block peptidoglycan polymerization via binding to the terminal D-ala-D-ala on the pentapeptide units (2). However, dalbavancin has some unique structural features compared with more thoroughly studied, related antibiotics such as teicoplanin, vancomycin (glycopeptides), and daptomycin (lipopeptide) (2, 49). We used CRISPRi-seq to get further insights into factors that affect susceptibility to dalbavancin (Tables S16 and S17). The minimal inhibitory concentration (MIC) of dalbavancin against *S. aureus* NCTC8325-4 carrying the CRISPRi system (strain AHF10) was found to be 0.06 µg/mL. The induced CRISPRi library was then grown in the presence and absence of 0.015 µg/mL dalbavancin for 12 generations to identify sgRNAs that were over- or under-represented after dalbavancin treatment (Fig. 4A). In total, 27 genes were found to have a significant reduction in fold change (|log_2_FC| > 1, *P*_adj_ < 0.05) in the presence of dalbavancin, indicating that knockdown of these genes results in increased sensitivity (Table 1; Fig. 4B), whereas 11 genes were found to have a significantly increased fold change under these conditions, suggesting that repression of these genes could cause reduced sensitivity to dalbavancin (Table 1; Fig. 4B).

**TABLE 1:** Genes affecting dalbavancin susceptibility as identified by CRISPRi-seq^*a*^

CRISPRi target locus	Target genes	Function	log_2_FC	*P*_adj_	MIC
**SAOUHSC_00890**	***kapB***	**Unknown**	**−3.7**	**3.0E−47**	**0.045**
SAOUHSC_01389	*pstS*	ABC transporter substrate-binding protein	−2.5	3.0E−27	0.06
SAOUHSC_02810	*SAOUHSC_02810*	Unknown	−2.3	5.4E−23	0.06
**SAOUHSC_01827**	***ezrA***	**Septation ring formation regulator**	**−3.0**	**1.5E−20**	**0.03**
**SAOUHSC_00867**	***_00867;dltXABCD***	**D-alanylation of teichoic acids**	**−4.1**	**2.5E−19**	**0.03**
**SAOUHSC_02447**	***_02447***	**Unknown**	**−2.0**	**2.5E−12**	**0.045**
**SAOUHSC_00667**	***vraFG***	**ABC transporter**	**−2.4**	**4.7E−12**	**0.03**
**SAOUHSC_00892**	***_00892***	**Unknown**	**−2.2**	**6.2E−12**	**0.045^*b*^**
**SAOUHSC_00646**	***pbp4***	**Penicillin-binding protein 4**	**−1.9**	**7.1E−12**	**0.03**
**SAOUHSC_00678**	***_00678***	**Unknown**	**−2.1**	**4.3E−09**	**0.045**
SAOUHSC_01055	*imp*	Inositol monophosphatase family protein	−1.8	1.2E−08	n.t.
**SAOUHSC_02383**	***facZ***	**EVE-domain protein**	**−3.0**	**3.3E−06**	**0.045**
**SAOUHSC_00743**	***nrdF***	**Ribonucleotide-diphosphate reductase**	**−2.4**	**5.9E-06**	**0.045^*b*^**
SAOUHSC_00472	*prs*	Ribose-phosphate pyrophosphokinase	−2.2	3.3E**−**05	0.06
SAOUHSC_00574	*eutD;lipL*	Phosphate acetyltransferase_N-octanoyltransferase	−2.0	5.8E**−**05	n.t.
SAOUHSC_01866	*ccrZ*	Cell cycle regulator	−1.7	0.00017	n.t.
SAOUHSC_02444	*opuD2*	BCCT-family osmoprotectant transporter	−1.8	0.00018	n.t.
SAOUHSC_01466	*recU;pbp2*	Holliday junction resolvase; penicillin-binding protein 2	−1.6	0.00039	n.t.
SAOUHSC_01548	*_01548*	Conserved hypothetical protein	−1.5	0.00040	n.t.
SAOUHSC_00833	*_00833*	Nitroreductase domain protein	−1.7	0.00070	n.t.
SAOUHSC_00663	*_00663*	N-acetyltransferase domain-containing protein	−1.7	0.00070	n.t.
SAOUHSC_01490	*hup*	DNA-binding protein HU	−1.8	0.00071	n.t.
SAOUHSC_00347	*_00347*	Unknown	−1.4	0.00196	n.t.
SAOUHSC_01252	*rnjB*	Ribonuclease J2	−1.7	0.00436	n.t.
SAOUHSC_02740	*_02740*	Drug transporter	−1.5	0.02159	n.t.
**SAOUHSC_00348**	***rpsF;ssb;rpsR***	**Ribosomal; ssDNA-bind; ribosomal**	**−2.2**	**0.04321**	**0.045^*b*^**
SAOUHSC_02459	*_02459*	Unknown	−1.3	0.04388	n.t.
SAOUHSC_02860	*mvaS*	HMG-CoA synthase	2.2	0.00016	0.06
SAOUHSC_01501	*ebpS*	Cell surface elastin binding protein	2.2	2.52E**−**10	0.09
**SAOUHSC_00659**	**_00659**	**Unknown**	**2.1**	**3.78E−08**	**0.09^*b*^**
SAOUHSC_01627	_01627;_01628	Unknown	1.9	0.00015	n.t.
SAOUHSC_02012	*sgtB*	Monofunctional glycosyltransferase	1.9	3.78E**−**08	0.06
SAOUHSC_A00332	_A00332	Unknown	1.9	1.76E**−**07	n.t.
SAOUHSC_00579	*mvaK2*	Phosphomevalonate kinase	1.9	0.00978	0.06
SAOUHSC_00531	_00531	Unknown	1.7	0.00040	n.t.
SAOUHSC_00685	_00685;_00686	Transcription factor; unknown	1.7	0.12676	0.06
SAOUHSC_00268	*esaD*	Type VII secretion system EssD	1.7	1.66E**−**20	n.t.
SAOUHSC_01960	*hemY*	Protoporphyrinogen oxidase	1.6	0.00026	n.t.

^*a*^ MIC-values are given in µg/mL. The MIC of the CRISPRi no-target control was 0.06 µg/mL. Experimentally verified hits are highlighted in bold. n.t., not tested.

^*b*^ Essential genes affecting dalbavancin susceptibility.

**Fig 5:**
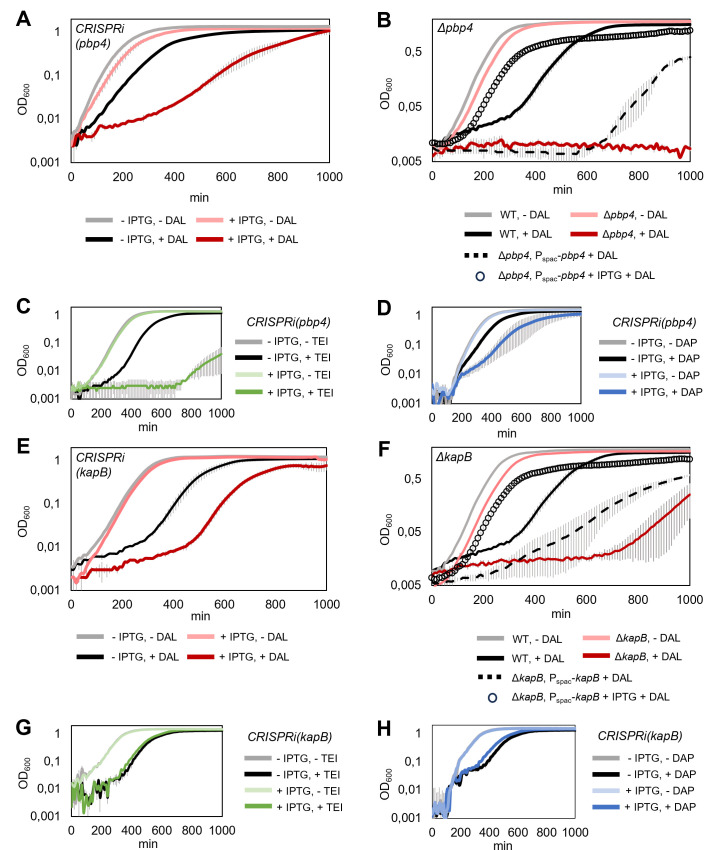
Contribution of *pbp4* and *kapB* to susceptibility of *S. aureus* toward dalbavancin, teicoplanin, and daptomycin. (**A–D**). The presence of functional *pbp4* results in increased tolerance to dalbavancin (**A and B**), teicoplanin (**C**), and daptomycin (**D**), as shown by knockdown (**A, C, D**) or deletion (**B**) of *pbp4*. Complementation of Δ*pbp4* with plasmid pLOW-P_spac_-*pbp4* grown in the presence of dalbavancin is shown in panel **B**. The dalbavancin sensitivity of the uninduced strain (dashed line) is reduced by induction of *pbp4* with 300 µM IPTG (black circles). (**E–H**). *kapB* influences susceptibility to dalbavancin, but not to teicoplanin or daptomycin as shown by knockdown (**E, G, H**) or deletion (**F**) of *kapB*. Complementation of Δ*kapB* with plasmid pLOW-P_spac_-*pbp4* grown in the presence of dalbavancin is shown in panel **F**. The dalbavancin sensitivity of the uninduced strain (dashed line) is reduced by induction of *kapB* with 300 µM IPTG (black circles). The antibiotic concentrations used were 0.03 µg/mL for dalbavancin and 1 µg/mL for teicoplanin and daptomycin. Averages of triplicates with standard errors are shown.

We created a selection of 13 individual CRISPRi strains expected to have an increased sensitivity to dalbavancin and tested their susceptibility to dalbavancin in 2-fold dilution assays in microtiter plates. Indeed, 10 of 13 strains tested displayed increased susceptibility to dalbavancin in these assays, with a maximum 2-fold MIC reduction (Table 1). These include not only genes or operons of known function, such as *dltABCD, vraFG*, *ezrA*, *rpsF*-operon, *nrdF,* and *pbp4* (Fig. 4C, D, E, F, I, and J and 5A) but also yet uncharacterized genes (e.g., SAOUHSC_00678, SAOUHSC_00892, and *kapB*, Fig. 4G and H and 5E). Importantly, the verified hits identified in this screen include genes that could not have been identified with any gene knockout approach due to their essentiality (Fig. 4B). For example, downregulation of *nrdF*, encoding a ribonucleotide-diphosphate reductase, SAOUHSC_00892, a putative RNA-binding protein of unknown function, as well as the ribosomal operon *rpsF-ssb-rpsR*, led to increased sensitivity to dalbavancin (Fig. 4C through J). Also, *facZ*, a recently identified gene important for Z-ring placement and proper cell division in *S. aureus* (50), was shown to have the same effect (Table 1). To further corroborate the results of the screen, we also created deletion mutants of the two non-essential genes *kapB* and *pbp4*. Indeed, similar to the knockdowns (Fig. 5A and E), the deletion mutants (Fig. 5B and F) displayed increased susceptibility to dalbavancin, with a 2-fold reduced MIC. The dalbavancin sensitivity of the *pbp4* and *kapB* deletion mutants was, as expected, reduced by complementing the mutants with plasmid-based, IPTG-inducible expression of the respective genes (Fig. 5B and F).

### KapB influences dalbavancin, but not other glycopeptide or lipopeptide antibiotic susceptibility

Several hits identified by the CRISPRi-seq screen have previously been linked to glycopeptide or lipopeptide antibiotic susceptibility like vancomycin and daptomycin. Such hits include the *dltABCD* operon (51), *vraFG* (5), *ezrA* (5, 51), SAOUHSC_00678 (5), and *pbp4* (5). The *dltABCD* operon (Fig. 4D) is responsible for modifying cell surface charge by D-alanylation of teichoic acids. The *vraFG* (for vancomycin resistance-associated F and G) operon (Fig. 4E) encodes a putative ABC transporter system and was reported as an important factor for vancomycin and daptomycin resistance (5). EzrA is the cell division and septum formation regulator (Fig. 4F), and mutants in this gene have previously been shown to be hypersensitive toward daptomycin (5, 51). The uncharacterized gene SAOUHSC_00678 (Fig. 4G) has previously been found to be associated with susceptibility to daptomycin, although its function is unknown (5). Finally, a connection between daptomycin or vancomycin sensitivity and *pbp4* has also been reported in some strains (5). To test whether the confirmed hits were general factors affecting susceptibility to glycopeptide or lipopeptide antibiotics, we exposed individual CRISPRi mutants to teicoplanin or daptomycin. Indeed, most of the hits, including *pbp4* (Fig. 5A, C, and D), also displayed increased sensitivity to antibiotics other than dalbavancin. Exceptionally, knockdown or knockout of *kapB* (“kinase-associated protein B,” encoding a non-essential protein of unknown function) led to a significant decrease in tolerance toward dalbavancin, but not toward the other antibiotics (Fig. 5E, G, and H). These results show that dalbavancin has a unique susceptibility determinant, suggesting that its mechanism of action differs from that of related antibiotics.

### Depletion of *ebpS* and SAOUHSC_00659 reduces the susceptibility to dalbavancin

In total, 11 hits had a significant positive log_2_ fold change upon dalbavancin treatment in the CRISPRi-seq experiment (Table 1; Fig. 4B), suggesting that depletion of these genes would result in reduced sensitivity. We selected five of the top hits (*ebpS*, *mvaS*, *mvaK2*, *sgtB*, and SAOUHSC_00659) for further verification with single depletion strains, and knockdown of two of these targets (*ebpS* and SAOUHSC_00659) was confirmed to increase tolerance to dalbavancin. The strains with depleted SAOUHSC_00659 or *ebpS* grew better than the control in the presence of dalbavancin (Fig. S5). We also noted that the depletion of EbpS resulted in a higher maximum OD in the stationary phase. The three remaining selected candidate genes did not differ from the control. SAOUHSC_00659 encodes a protein of unknown function, whereas *epbS* encodes a surface-exposed, integral membrane protein known as elastin binding protein (52). The functions of these two proteins are largely unknown, although EbpS has been reported to bind elastin *in vitro* (52, 53) and has also been suggested to be an important factor for biofilm formation under certain conditions (54). Our study suggests that EbpS and SAOUHSC_00659 are somehow involved in a response to cell envelope stress caused by dalbavancin.

### Direct selection of dalbavancin-tolerant strains reveals that the shikimate pathway modulates dalbavancin tolerance

**Fig 6:**
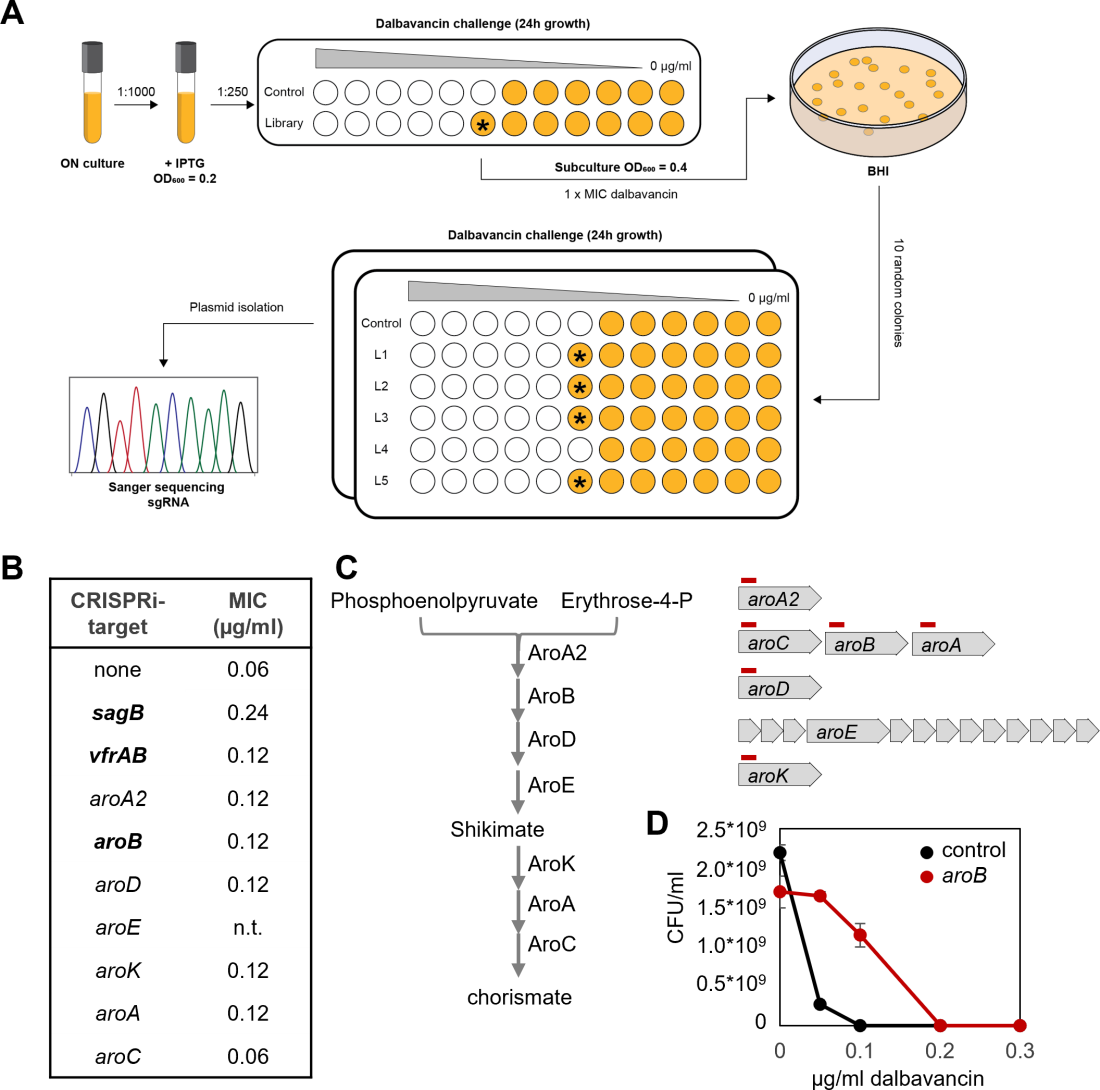
Direct selection of dalbavancin tolerant strains. (**A**) Schematic outline of the CRISPRi library selection study for dalbavancin susceptibility. The asterisks point to wells with increased growth compared with the controls. (**B**) List of hits for verification studies. The three hits identified by the CRISPRi library selection study are indicated in bold. (**C**) Schematic illustration of Shikimate pathway (left) and corresponding gene arrangement on the genome (right). The red lines indicate binding sites of the sgRNAs. (**D**). Population analysis profile showing that the *aroB* knockdown strain survives at higher concentrations of dalbavancin compared with the control.

Besides the CRISPRi-seq screen, the CRISPRi library holds potential in enrichment and selection studies under specific stresses, apart from next-generation sequencing. As a complementation for the CRISPRi-seq screens of dalbavancin-related factors, we used the CRISPRi library to select for strains with reduced susceptibility to dalbavancin as outlined in Fig. 6A (see also Materials and Methods). The sgRNAs harbored by eight colonies shown to display increased dalbavancin susceptibility were identified by Sanger sequencing (Fig. 6A). Five of the strains carried sgRNAs targeting *sagB* (SAOUHSC_01895), two *aroB* (SAOUHSC_01482), and one *vrfA* (SAOUHSC_01192). To verify that the increased resistance was not caused by any secondary mutations in the genome, the sgRNA plasmids were re-introduced into NCTC8325-4 to make new, individual CRISPRi mutants. Dalbavancin susceptibility of these mutants was tested, and the results confirmed that knockdown of these genes resulted in reduced dalbavancin sensitivity (Fig. 6B).

Knockdown of *sagB* led to a 4-fold increase in MIC to dalbavancin (Fig. 6B). *sagB* encodes a glucosaminidase involved in peptidoglycan polymer length control in *S. aureus* (55, 56). Consistent with our discovery, mutations in *sagB* were previously reported to reduce sensitivity to vancomycin due to the extended peptidoglycan polymers present in the mutant (55). The reduced sensitivity to dalbavancin might be caused by the same mechanism. The *vfrA*-gene, whose function is unknown, is encoded on the same operon as *fakA* (formerly known as *vfrB*). FakA encodes a fatty acid kinase that is needed for utilization of exogeneous fatty acids for lipid synthesis. Deletion of *fakA* has been shown to alter the metabolic state of the cell, including changes in intracellular amino acid concentrations (57). AroB encodes the 3-dehydroquinate synthase in the Shikimate pathway for biosynthesis of the amino acid precursor chorismate (Fig. 6C), and knockdown of *aroB* was confirmed to increase dalbavancin tolerance (Fig. 6D). The link between chorismate biosynthesis and susceptibility to glycopeptides has not been reported before. To further verify these results, CRISPRi strains to knock down other genes in the Shikimate pathway were constructed: *aroA2*, *aroC*, *aroA*, *aroD*, and *aroK* (Fig. 6C). The dalbavancin MIC was determined, and indeed, the MIC increased slightly in all strains except for *aroC* (Fig. 6B), showing that disruption of the Shikimate pathway modulates susceptibility to dalbavancin in *S. aureus* NCTC8325-4. The deviating result for the *aroC* CRISPRi strain may be caused by the low efficiency of this specific sgRNA, since other strains targeting genes in the same operon (*aroB* and *aroA*) clearly affect the sensitivity.

## DISCUSSION

In this work, we have developed a pooled, genome-wide, compact operon-based CRISPRi library with 1,928 sgRNAs for *S. aureus* (Fig. 2). To enable high-throughput genetic screening, we combined the CRISPRi library with Illumina sequencing and developed CRISPRi-seq in *S. aureus*. In contrast to the previously developed *S. aureus* CRISPRi library, which was made by a CRISPR adaptation strategy and contains an average of 100 guide RNAs per gene (22), the compactness of our library with one systematically designed sgRNAs per gene requires less sequencing depth and could more easily overcome bottlenecks in CRISPRi-seq experiments, which may occur in some experimental settings such as in animal models (16). Initially, we employed CRISPRi-seq to quantify gene fitness in two *S*. *aureus* strains, NCTC8325-4 and Newman (Fig. 3). By comparative analysis of the genes whose repression resulted in a significant fitness defect in these two strains by CRISPRi-seq with published essentialome data, we showed that CRISPRi-seq could capture the conserved essential genes across different *S. aureus* strains. Approximately 75% of the sgRNAs expected to target conserved essential genes according to transposon-based knockout studies in related *S. aureus* strains (40–42) indeed had significantly reduced abundance in the CRISPRi-seq screen. Nevertheless, a significant fraction (19%) of the conserved essential genes was not defined as essential in our CRISPRi-seq results. This may be explained by different strains used and differences in growth conditions between the studies: gene essentiality is strain- and condition-specific. Alternatively, some of these missing hits may represent false negatives, resulting from sgRNAs with poor efficiency or insufficient depletion during the experiments. Lastly, differences between studies may be partially explained by differences in the definition of essentiality, as in our studies, we used relatively strict statistical parameters for this designation (significantly more than doubling or halving of the CRISPRi strain abundance upon CRISPRi induction), and transposon mutagenesis-based studies requires different statistical approaches to determine essentiality.

It is expected that the number of genes with significant low fitness scores would increase with the number of generations of growth during CRISPRi induction. Two independent CRISPRi-seq experiments (12 and 20 generations of growth) were performed in this study; however, there were very few differences between the two experiments (Fig. 3). Thus, 12 generations of growth are sufficient to capture most target genes with major fitness effects in these experimental conditions. Furthermore, our *in silico* analysis suggests that the sgRNA library is largely functional in other, commonly used *S. aureus* strains (64% to 89% of sgRNAs with an exact non-template strand within-gene binding site), covering most of their annotated genetic elements either directly, by binding to the annotated gene (53% to 63%), or, indirectly, by polar effects (79% to 93%) (Fig. S1; Tables S4 to S10). Note that the former percentages are based on actual annotations, that is, they are measured, whereas the latter are estimated by extrapolation, assuming similar operon structures between all strains and NCTC-8325-4 (as defined by reference 38). It is possible, however, that these estimates are still on the conservative side, as differences in annotations might hinder target gene detection and sgRNAs might still be active at sites with minor allelic differences between homologs, as long as the mismatches are few and on the PAM-distal side of the sgRNA spacer. Although we have indeed shown the library’s functionality in one such strain, Newman (Fig. S4), it should be noted that its use in any other strain will be inferior to NCTC8325-4, which the library was designed for.

We next explored genes related to dalbavancin susceptibility using CRISPRi-seq and CRISPRi-oriented selection studies (Fig. 4 and 6). Dalbavancin is a semi-synthetic lipoglycopeptide with broad activity against Gram-positive bacteria, including *S. aureus* MRSA strains (25). To get further insights into the mechanism of action of dalbavancin and its unique features, we applied CRISPRi-seq under dalbavancin stress. Interestingly, we identified and validated both genes that led to increased and decreased dalbavancin tolerance. We found many genes known to be involved in susceptibility to vancomycin (glycopeptide), teicoplanin (vancomycin-derivative), or daptomycin (lipopeptide) to also influence dalbavancin sensitivity. This indicates that there are similar mechanisms contributing to resistance and sensitivity for these antibiotics, which is unsurprising, given that they all target the cell wall and/or membrane.

However, we also showed that a new factor, *kapB*, encoding a 127 aa conserved protein, specifically contributes to *S. aureus* susceptibility toward dalbavancin, but not other glycopeptides or lipopeptides (Fig. 5), suggesting that dalbavancin has an antibacterial mechanism that, to some extent, differs from teicoplanin and related antibiotics. The function of KapB and how it affects dalbavancin sensitivity remains to be fully explored. *kapB* is monocistronic and located between the *mnhABCDEFG* operon and *ppi*, encoding a multicomponent monovalent cation/H+ antiporter system and a putative peptidyl-prolyl cis-trans isomerase, respectively. Knockdown of neither of these neighboring genes had any significant impact on dalbavancin susceptibility in the CRISPRi-seq screen, suggesting that KapB acts independently (Table S17).

Furthermore, in our assays, *S. aureus* NCTC8325-4 mutants lacking PBP4 became more susceptible to dalbavancin and teicoplanin (Fig. 5). PBP4 is a transpeptidase in *S. aureus,* important for the formation of cross-links in *S. aureus* peptidoglycan. In contrast to our findings here, inactivation or reduced expression of *pbp4* has previously been linked to glycopeptide-resistant mutants, which has suggested a mechanism where resistance is caused by the accumulation of non-crosslinked muropeptides in these mutant strains, resulting in binding and sequestering of the antibiotics (58, 59). However, in contrast to the NCTC8325-4 strain, glycopeptide-resistant mutants often carry multiple, diverse mutations contributing to the resistance phenotype (35, 60). Moreover, previous experiments with Δ*pbp4* mutants in glycopeptide-sensitive strains showed that the vancomycin MIC is the same as for their respective wild type (59, 61, 62). We also confirmed that this was the case for NCTC8325-4 (MIC of 2 µg/mL vancomycin for both wild type and Δ*pbp4*). In addition, it was recently reported that a *pbp4* deletion increased the susceptibility to vancomycin in a bone tissue infection model (62). The association between PBP4 and glycopeptide antibiotics in different genetic backgrounds should therefore be further investigated, particularly since dalbavancin has been shown to act synergistically with different β-lactam antibiotics (63–66).

Knockdown of either SAOUHSC_00892, *nrdF,* or *rpsF-ssb-rpsR* led to increased sensitivity to dalbavancin in the CRISPRi-seq screen. These genes have not previously been identified as contributors to antibiotic susceptibility by any knockout-based screening due to their essentiality for growth, demonstrating that CRISPRi-seq can be used as an important complementary technique for high-throughput screenings in *S. aureus*. The molecular mechanisms explaining why these genes influence dalbavancin susceptibility remain to be elucidated.

We also used a direct selection approach to screen for strains with reduced sensitivity to dalbavancin (Fig. 6) and found other genes (*sagB*, *aroB,* and *vfrAB*) than by CRISPRi-seq (Fig. 4), demonstrating that the output of such screens depends on the experimental setup. Indeed, the hits identified in the direct selection approach were not defined as having significantly differential fitness by the CRISPRi-seq approach (Table S17). Of these hits, SagB, a glucosaminidase cleaving strands of peptidoglycan, has previously been shown to result in reduced vancomycin sensitivity (55). The *vfrA-fakA* operon encodes proteins with similarity to acid shock protein Asp23 and a fatty acid kinase, respectively. The role of VfrA is unknown, but previous studies have shown that FakA is responsible for the incorporation of exogeneous fatty acids into phospholipids *in vivo* (67). Notably, mutations in *fakA* have indeed been identified in dalbavancin-resistant mutants generated by antibiotic exposure (36). Of note, we also demonstrate that knocking down the Shikimate pathway can result in reduced sensitivity to dalbavancin *in vitro* (Fig. 6). The Shikimate pathway has been considered a promising target for antibacterial drugs because it has no counterpart in mammals and is essential for bacterial growth and virulence in many contexts (68). However, the results shown here argue against Shikimate pathway genes as therapeutic targets, as their depletion may also reduce antibiotic susceptibility.

In this work, we have created a tight, compact CRISPRi library and showed its functionality and utility in *S. aureus* in both CRISPRi-seq and direct selection experiments. We have uncovered genes previously unknown to be involved in antibiotic susceptibility. Among these are core essential genes that cannot be detected using more traditional mutagenesis approaches as such mutants are, by definition, excluded from those libraries due to their inability to grow. This study will serve as a steppingstone to explore the molecular mechanisms involved in antibiotic tolerance in general and of dalbavancin in particular.

## MATERIALS AND METHODS

### Bacterial strains and growth conditions

*S. aureus* strains were grown in BHI or TSB medium at 37°C with shaking or on BHI agar plates. When needed, 5 µg/mL erythromycin and/or 10 µg/mL chloramphenicol were added for selection. Transformation of *S. aureus* was performed with electroporation as described before (69). *E. coli* was grown on lysogeny agar (LA) agar or in lysogeny broth (LB) medium at 37°C with shaking. Ampicillin (100 µg/mL) was added for selection. *E. coli* was transformed using heat shock of chemically competent cells. A list of all strains used in the study is found in Table S18.

### Plasmid and strain construction

#### pLOW-P_spac2_-dcas9

The P*_spac_* promoter of the pLOW-plasmid used to construct our original CRISPRi system contains a single *lacO* operator site (12, 37). An extra *lacO* operator site was introduced 58 nt upstream of the pre-existing *lacO*-operator to create the promoter P*_spac2_*. The position of the *lacO* site was selected based on the P_spac_ promoter in plasmid pLA8-1, which is tightly regulated with a high dynamic range in *S. pneumoniae* (70). The *lacO* site was introduced by inverse PCR, using primers mvh54_Pspac_Rev and mvh55_intro_lacO_Pspac with pLOW-*dcas9* as template. The product was digested with DpnI and transformed into *E. coli* IM08B. Correct transformants were verified by PCR and sequencing. The primers used in this study are listed in Table S19.

#### pVL2336

The plasmid expressing the sgRNAs, pCG248-sgRNA(*xxx*) (12), was modified to allow Golden Gate cloning and library preparation for Illumina sequencing. The two BsmBI sites on the vector were removed by circular PCR with OVL2127 and OVL2128 followed by BsmBI digestion and T4 self-ligation. After that, the vector pCG248-sgRNA(*xxx*) was linearized by digestion with BglII and BamHI; and a DNA fragment containing read1 (Illumina sequencing element), P3 (constitutive promoter for sgRNA expression), BsmBI site (producing cut-edge at +1 of P3), mCherry (as reporter for the cloning), second BsmBI site (producing cut-edge between the base pairing region and dCas9 handle binding region), and read2 (Illumina sequencing element) was amplified from pPEPZ-sgRNAclone (Addgene# 141090) with OVL2152 and OVL2153 (23). The amplified DNA fragment was then cloned into pCG248-sgRNA(*xxx*) to replace the pCG248-sgRNA(*xxx*) fragment by infusion cloning, resulting in the sgRNA cloning vector pVL2336.

#### Construction of single sgRNA plasmids

New 20 bp sgRNA sequences were cloned into pVL2336 using Golden Gate cloning as described before (16). Briefly, two oligos for each sgRNA (Table S20) were diluted in TEN buffer (10 mM Tris, 1 mM EDTA, 100 mM NaCl, pH8), incubated at 95°C for 5 min and then slowly cooled down to room temperature for annealing. The vector pVL2336 was digested with BsmBI to remove the mCherry fragment and purified by gel extraction. The purified vector and the annealed oligos were ligated using T4 ligase and transformed into *E. coli* IM08B. Correct vectors were verified by Sanger sequencing with primer MK25.

#### Construction of complementation plasmids pLOW-pbp4 and pLOW*-*kapB

*pbp4* was amplified using primers mk619 and mk620, whereas *kapB* was amplified using primers mk621 and mk622. The plasmid vector pLOW-dcas9 and the fragments were digested with SalI and NotI, and the fragments were ligated into the pLOW-vector downstream of the P_spac_-promoter and transformed into *E. coli* to produce pLOW-pbp4 and pLOW-kapB. The correct vectors were checked by PCR and sequenced prior to transformation into *S. aureus*.

### Construction of deletion mutants (Δ*pbp4*::*spc* and Δ*kapB*::*spc* mutants) in *S. aureus* NCTC8325-4

pMAD-pbp4::spc was assembled using overlap extension PCR and ligated into pMAD using restriction cloning. The region upstream and downstream of *pbp4* were amplified with primer pairs mk411/mk412 and mk413/mk414, respectively. The spectinomycin resistance cassette was amplified with primer pair mk188/mk189. The inner primers mk412 and mk413 contain overlapping sequences, allowing fusion of the fragments in a second step PCR to make the fusion construct *pbp4*_up-*spc-pbp4*-down. The fused fragment was digested with NcoI and BamHI, whose restriction sites were introduced with the outer primers mk411 and mk414, respectively, and ligated into the corresponding sites of pMAD. The ligated plasmid was transformed into *E. coli* IM08B and verified by PCR and sequencing.

pMAD-kapB::spc was assembled and ligated into pMAD-GG (pMAD-plasmid adapted for Golden Gate cloning, laboratory collection) by Golden Gate cloning. The up- and down-stream regions were amplified with primer pairs AHF13/AHF14 and AHF17/AHF18, respectively. The spectinomycin cassette was amplified with AHF15/AHF16. BsaI restriction sites generating complementary overhangs are introduced in the primers. The purified fragments and the pMAD-GG vector were digested with BsaI and assembled using NEB Golden Gate Assembly kit. The assembly reaction was transformed into *E. coli* IM08B and verified by PCR and sequencing.

Following construction of the plasmids, the deletion strains were made as previously described for the temperature-sensitive pMAD system (71). Plasmids were transformed into *S. aureus* at a permissive temperature (30°C) and plated onto X-gal plates with erythromycin selection. Blue colonies were re-streaked at 30°C and verified by PCR. A single colony was picked in medium without selection and incubated at 30°C for 2 h before the tube was transferred to 43°C for 6 h. The culture was plated onto TSA plates with X-gal and spectinomycin for selection and incubated at 43°C. Candidates for double crossover (white colonies) were re-streaked on two separate plates to identify colonies that were spectinomycin-resistant and erythromycin-sensitive, and strains were further verified by PCR and sequencing.

### CRISPRi library construction

#### Design of sgRNAs

The coding sequences of the selected genes were used as input for PAM motif search using CRISPR Primer Designer (72). The 20 bp sequence adjacent to the 5′ proximal PAM, targeting the non-template strand, was selected as the candidate sgRNA target sequence. To check the specificity of the 20 bp sequences, the core 12 bp sequence adjacent to the PAM (core sgRNA) was used for a Basic Local Alignment Search Tool (BLAST) search, using the NCTC8325 genome as a target, to filter out non-specific targets. The 20 bp sequence closest to the 5′ end of the gene fulfilling these criteria was selected as the final target. Finally, 1,928 sgRNAs were designed (Tables S1 and S20).

#### Construction of the genome-wide sgRNA library in *E. coli* IM08B

The 20 bp base-pairing region of the sgRNAs was first cloned into pVL2336 in *E. coli* IM08B by Golden Gate cloning. A total of 1.3 × 10^6^ transformant colonies were obtained, providing a 674-fold coverage of the 1,928 sgRNAs library. The false-positive ratio of sgRNA cloning was estimated by calculating the percentage of the red colonies among all transformant colonies, which showed a false-positive rate lower than 0.03% (no red colony was identified among 3,000 colonies, as determined by visual inspection). The plasmids carrying the sgRNAs were then purified from *E. coli* IM08B and used as a template in the one-step PCR for Illumina amplicon library preparation as previously described (23).

### Construction of CRISPRi library in *S. aureus* NCTC8325-4 and Newman

Plasmids isolated from the *E. coli* sgRNA library were transformed into *S. aureus* MH225 (NCTC8325-4, pLOW-Pspac2-dcas9) and *S. aureus* MH226 (Newman, pLOW-Pspac2-dcas9) by electroporation. Multiple parallel transformation reactions were plated onto BHI agar plates containing 5 µg/mL erythromycin and 10 µg/mL chloramphenicol. More than 200,000 colonies were collected using a cell scraper, pooled and resuspended in 100 mL BHI containing 5 µg/mL erythromycin and 10 µg/mL chloramphenicol. The pooled libraries were diluted (1.5 mL in 250 mL Erlenmeyer flasks containing 100 mL BHI with 5 µg/mL erythromycin and 10 µg/mL chloramphenicol) and incubated at 37°C with shaking (180 rpm) until OD_600_ = 0.8 and stored as glycerol stocks for further use.

### CRISPRi-seq screens

Growth of the library for CRISPRi-seq experiments was performed in 100 mL BHI medium supplemented with 5 µg/mL erythromycin and 10 µg/mL chloramphenicol for selection using 250 mL Erlenmeyer flasks. Cultures were grown with shaking at 37°C. When appropriate, 250 µM IPTG was added for induction. Four parallels per condition were used. For the 20-generation experiment, the library was inoculated 1/1,000 from the stock, grown until OD_600_ = 0.8, then reinoculated 1/1,000 in fresh medium and again grown until OD_600_ = 0.8. The cultures were then transferred to 50 mL Nunc tubes and collected by centrifugation (6,000 × *g*, 5 min, 4°C). For the 12-generation experiment, the library was inoculated 1/4,000 from the stock and grown to OD_600_ = 0.8 before cells were harvested. For the dalbavancin experiment, the same conditions as in the 12-generation experiment were used, except that a sublethal concentration (0.015 µg/mL, equivalent to one-fourth of the MIC) was added. For plasmid isolation, cells were lysed by treatment with lysostaphin (40 µg/mL, 30 min at 37°C) using QIAGEN Plasmid Midi Kit.

### Library preparation and Illumina sequencing

For the 12-generation experiment, construction of the amplicon library for Illumina sequencing was performed as described in our previous study (23). Specifically, plasmids were purified from the *S. aureus* CRISPRi library samples. Concentration of the plasmids was quantified with Nanodrop. To prepare the Illumina amplicon library, 1 µg of plasmids was used as a template for the one-step PCR with primers described before (23). The PCR products were purified by gel extraction. The purified products were then quantified by Qubit and processed for MiniSeq sequencing with a custom recipe as described previously (23).

For the 20-generation experiments, the Illumina amplicon library to be sequenced was amplified using the condition described previously (73). Briefly, 2 µL of the plasmid was used as a template and amplified using the Nextera DNA Indexes (Illumina Inc., San Diego, CA, USA) index primers. The PCR product was then purified and normalized using SequalPrep Normalization Plate Kit (Thermo Fisher Scientific, Waltham, MA, USA). The purified library was quantified using the KAPA library quantification kit (KAPA Biosystems, Wilmington, MA, USA) and sequenced on an Illumina Miseq platform (Illumina Inc) using the Miseq Reagent Kit v3 (Illumina Inc.).

### CRISPRi-seq differential enrichment analyses

Read pairs of the paired-end sequencing data of the NCTC8325 20-generation experiment and the sequenced plasmid pool were merged using PEAR (v0.9.11) (74) prior to sgRNA count extraction with 2FAST2Q (v2.5.0) (75) as for the other (single-end) sequencing data. Because of poor sequencing quality on the read extremes for the single-end sequencing data, alignment with 2FAST2Q was performed using the 2–17 nucleotides of the sgRNA sequences for those files. Otherwise, 2FAST2Q was used with default parameters. sgRNA depletion/enrichment was tested using DESeq2 in R (v4.1.1) (76). We always tested against an absolute log_2_ FC of 1 at a significance level of α = 0.05. For principal component analyses, counts were normalized with DESeq2’s blind rlog transformation. Sample 9_S9_L001_R1_001 (screening for NCTC8325, 12 generations, uninduced, first replicate) showed aberrant read count patterns and did not correlate with its replicates; hence, it was excluded from further downstream analyses. For comparison of 12- and 20-generation experiments, log_2_ FC values were scaled, but not centered, through division by the root mean square of the values per experiment using the built-in scale() function in R.

### Evaluation of target efficiency of the sgRNA library

sgRNA library efficiency on several genomes was evaluated using a previously published pipeline: https://github.com/veeninglab/CRISPRi-seq (23). The script sgRNA_library_evaluation_cmd.R was run with default settings, and GenBank files as input. GenBank files with the following nine assembly accession numbers were obtained from the NCBI RefSeq database, accessed on 5 September 2022: GCF_000009005 (RF122), GCF_000009665 (Mu50), GCF_000010465 (Newman), GCF_000011505 (MRSA252), GCF_000012045 (COL), GCF_000013425 (NCTC8325), GCF_001027105 (DSM20231), GCF_002085525 (JE2-USA300), and GCF_900475245 (NCTC8325). The total numbers of annotated features (i.e., unique locus_tag flags) were extracted from the genbank files (2,743, 2,910, 2,930, 2,881, 2,792, 2,872, 2,761, 2,881, and 2,858, respectively) and used in combination with the pipeline summary output files to compute the percentage of features that were covered by direct binding of at least one sgRNA within the feature boundaries on the non-template strand, without mismatches. Indirect coverage was then estimated by extrapolation: according to the summary output file, 1,864 NCTC8325 (GCF_000013425) features were directly targeted. We know from the design phase that 2,764 of 2,836 features were targeted considering known operon structures and polar effects. We then estimated for each genome the number of indirectly targeted features as the number of direct targets multiplied by 2,764/1,864, assuming similar operon structures among the strains. Note that due to differences between the current RefSeq annotations and those used in the design phase, according to this estimate, the target genome is covered 96.2%, instead of the pre-computed 2,764/2,836 = 97.5%. Similarly, library functionality was computed as the percentage of sgRNAs that have a zero-mismatch binding site on the non-template strand within the boundaries of an annotated feature in each genome.

### Growth assay

Growth curves were monitored in 96-well microtiter plates. Overnight cultures were diluted 100-fold in fresh medium, and then, 200 µL of the diluted culture was added into each well of 96-well plate as the starting culture. The microtiter plates were incubated at 37°C with shaking for 3 seconds prior to each measurement. OD_600_ was measured every 10 min using Synergy H1 Hybrid Reader (BioTek) or Hidex Sense (Hidex Oy). When appropriate, erythromycin and chloramphenicol were added for selection, and IPTG was added for induction.

### Antibiotic susceptibility assays

Susceptibility to antibiotics (dalbavancin, teicoplanin, and daptomycin) in liquid medium was determined using 2-fold dilution assays in microtiter plates. Two-fold dilution series of the antibiotics were made in growth media containing erythromycin and chloramphenicol (for selection). When appropriate, IPTG was added for induction. Unless otherwise mentioned, 250 µM IPTG was used. For the dalbavancin assays, 0.002% Tween-80 was added to the medium. For daptomycin, the assays were performed in the presence of 0.05 mg/mL CaCl_2_. Exponentially growing cells were diluted 100-fold. The microtiter plates were incubated at 37°C with shaking for 3 seconds prior to each measurement, and OD_600_ was measured every 10 min using Synergy H1 Hybrid Reader (BioTek) or Hidex Sense (Hidex Oy). Growth and antibiotic susceptibility assays were repeated two or three times, as indicated in the figure legends.

### Population analysis profile

Population analysis profiles were performed as described (77). Briefly, cultures induced with 250 µM IPTG were grown to OD 0.8 and diluted 10^–4^ to 10^–7^. Dilutions were then plated on BHI plates containing dalbavancin (0, 0.1, 0.2, and 0.3 µg/mL). Plates were incubated at 37°C, and colonies were counted after 24 h.

### Selection of dalbavancin-tolerant mutants

Overnight cultures of the CRISPRi library and a control strain [AHF1010; pLOW-dcas9, pVL2336-sgRNA(nontarget)] were re-diluted 1:1,000 in BHI containing antibiotics for selection and 250 µM IPTG for induction and grown until OD_600_ = 0.2. Then, a 2-fold dilution of dalbavancin (starting from 4 µg/mL dalbavancin) was set up, 250-fold dilutions of the cultures were added, and the plates were incubated overnight. From the library, cells from the well with the highest concentration of dalbavancin allowing growth of the library were further inoculated in a medium containing 250 µM IPTG and 1× MIC dalbavancin (0.06 µg/mL) until OD = 0.4. From this culture, dilutions were plated onto TSB plates containing chloramphenicol and erythromycin to obtain single colonies. From two independent experiments, 10 random colonies were picked, and another microtiter assay was performed to determine whether the susceptibility to dalbavancin differed from the control. For strains with increased susceptibility, plasmids were isolated and the sgRNA was sequenced by Sanger sequencing using primer MK25 to identify which gene was depleted.

## Data Availability

All sequencing data generated in this study are available on SRA, accession number PRJNA994855.

## AFTER EPUB

*[This article was published on 5 June 2024 with a missing acknowledgment. The Acknowledgments were corrected in the current version, posted on 7 June 2024.]*

## SUPPLEMENTAL MATERIAL

Supplemental material msystems.01289-23-s0001.pdf Supplemental figures; Tables S18 and S19; legends to Tables S1-S17 and S20.

Additional supplemental tables msystems.01289-23-s0002.xlsx Tables S11-S17 and S20.

Supplemental tables msystems.01289-23-s0003.xlsx Tables S1-S10.

ASM does not own the copyrights to Supplemental Material that may be linked to, or accessed through, an article. The authors have granted ASM a non-exclusive, world-wide license to publish the Supplemental Material files. Please contact the corresponding author directly for reuse.
