# Supplementary material for: Genome-wide CRISPRi screens for high-throughput fitness quantification and identification of determinants for dalbavancin susceptibility in Staphylococcus aureus
Source: mSystems. 2024 Jun 5;9(7):e01289-23. doi: 10.1128/msystems.01289-23 (PMC11265419; doi:10.1128/msystems.01289-23)
Supplement: Supplemental material — Supplemental figures; Tables S18 and S19; legends to Tables S1-S17 and S20. [file msystems.01289-23-s0001.pdf]

Supplementary figures and tables

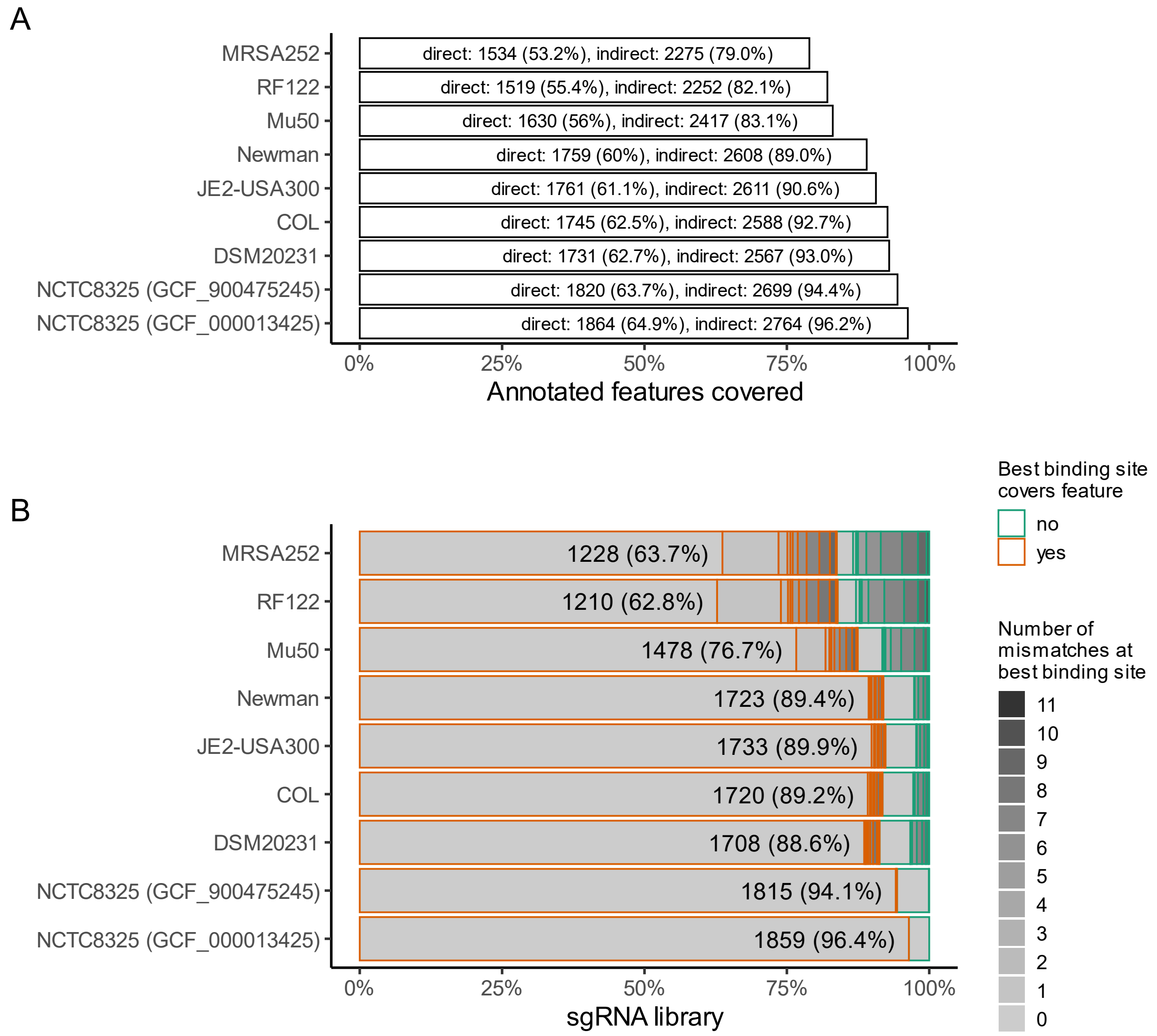

Fig S1. Library coverage across *S. aureus* genomes. (A) Genome coverage of the NCTC8325-based sgRNA library. Overview showing to which extent the library covers the genomes of various *S. aureus* strains. "Direct" indicates the number of features targeted by at least one sgRNA within the feature on the non-template strand, without mismatches. "Indirect" indicates the estimated number of features targeted, accounting for polar effects and operon structures. (B) Overview showing the proportion of the 1928 sgRNAs that are functional in various *S. aureus* strains. Note that two different annotations of the NCTC8325 genome are included (GCF_000013425 and GCF_900475245). For each sgRNA, the best binding site in the genome was identified using the library evaluation pipeline (see Methods). Bars indicate whether these binding sites are located within annotated features on the non-template strand, and the number of mismatches between the binding site and the sgRNA. Numbers are shown explicitly for sgRNAs targeting an annotated feature without mismatches. Any discrepancies between the actual target genome coverage/library functionality and the estimates reported here are due to discontinued and merged annotations in the current RefSeq version (GCF_000013425).

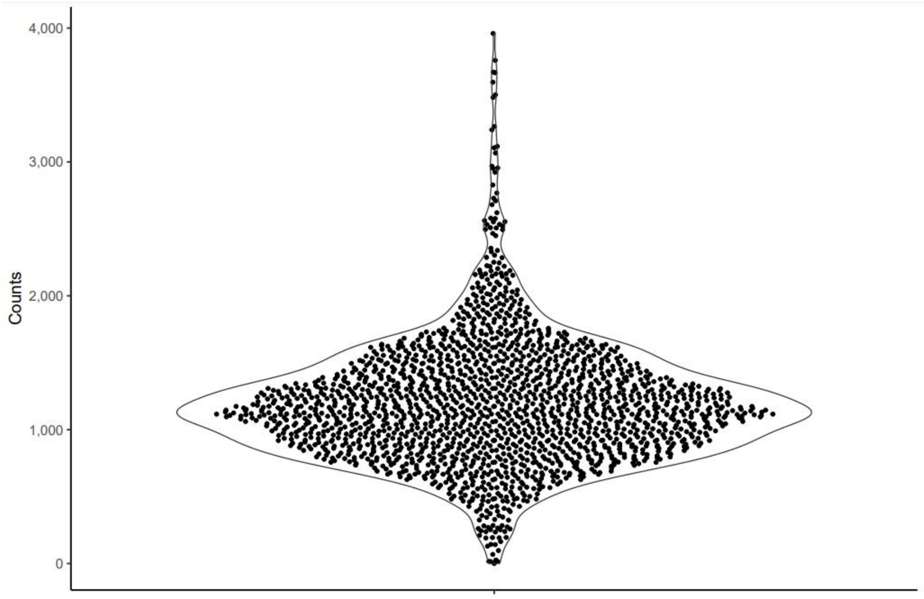

Fig. S2. Violin plot showing sgRNA distribution of the plasmids isolated from *E. coli* IM08B. Each dot represents one sgRNA.

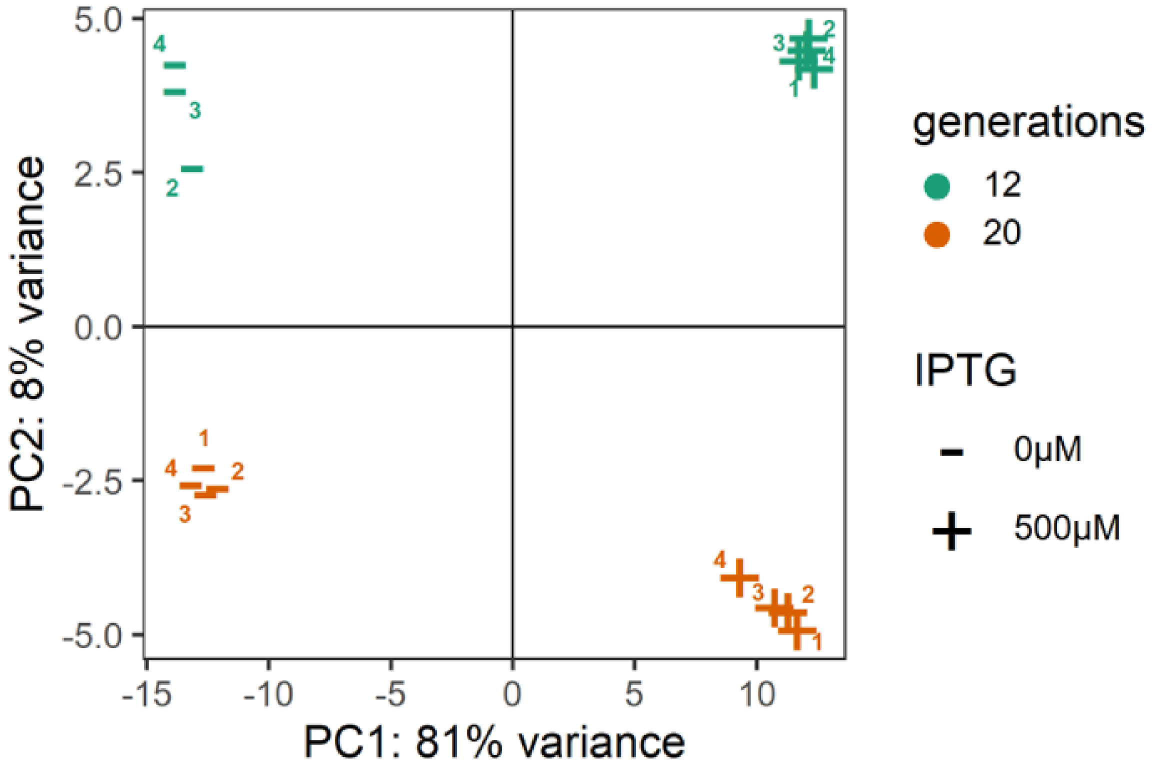

Fig. S3. Principal Component Analysis of the rlog-transformed (1) sgRNA counts. Replicate 1 of the uninduced 12-generation experiment samples was excluded, as many CRISPRi strains were depleted from it (without induction), yielding low correlations with the other replicates in the process.

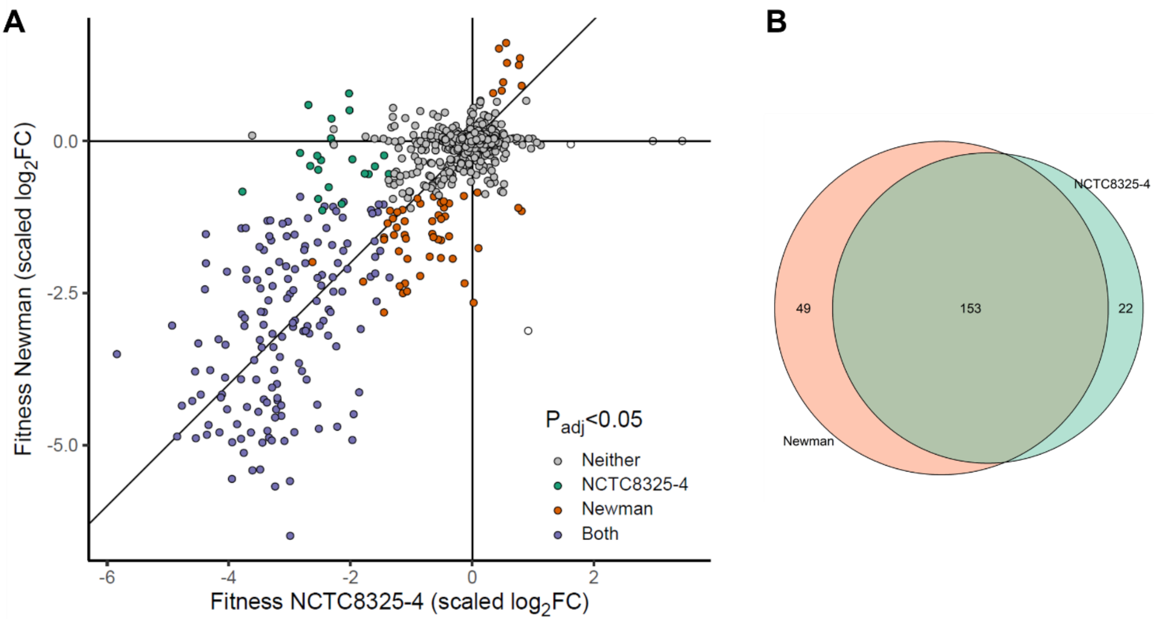

Fig. S4. CRISPRi-seq analysis of *S. aureus* Newman grown for 12 generations. (A) Genome-wide fitness comparisons between Newman and NCTC8325-4. The colors indicate sgRNAs that are significantly depleted or enriched in both strains (purple), neither (grey), only in Newman (orange) or only in NCTC8325-4 (green). Four sgRNAs lack color since their NCTC8325-4 p-value could not be computed due to count outliers as detected by DESeq2. (B) Venn diagram showing the overlap in sgRNAs with significantly reduced abundance determined by CRISPRi-seq in *S. aureus* Newman and NCTC8325-4.

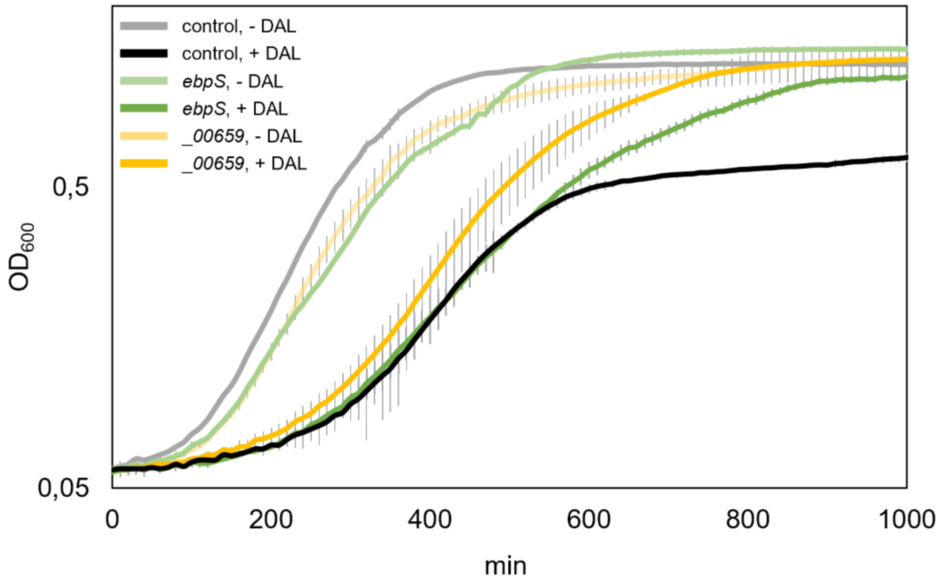

Fig. S5. Knockdown of *ebpS* and SAOUHSC_00659 led to reduced susceptibility to dalbavancin. CRISPRi control strain (no-target control, MM75), and CRISPRi strains targeting e*bpS* and SAOUHSC_00659 were all grown in the presence of 250 µM IPTG for induction of dCas9 expression. 0.03 µg/ml dalbavancin was added where indicated. Averages of triplicates with standard errors are shown.

Table S18. List of *S. aureus* strains in this study

Strain name	Genotype	Reference
E. coli		
IM08B	DH10B, Δ*dcm*, Phelp-*hsdMS*, PN25-*hsdS* (strain expressing the S. aureus CC8 specific methylation genes)	(2)
VL2336	Read1-P3-BsmBI-mCherry-BsmBI-dCas9 handle-Sp_{ter}-cat, Amp^R	This work
S. aureus		
NCTC8325-4	Derivative of NCTC8325, cured of prophages	(3)
Newman	Clinical isolate (ATCC 25904), rsbU+	(4)
MK1465	NCTC8325-4, pLOW-dCas9, Ery^R	(5)
MH225	NCTC8325-4, pLOW-P_{spac2}-dCas9, Ery^R	This work
MH226	Newman, pLOW2-P_{spac2}-dCas9, Ery^R	This work
MK1482	NCTC8325-4, GFP^+	This work
MH220	MK1482, pLOW-P_{spac2}-dCas9, pVL2336-sgRNA(gfp), Ery^R, Cam^R.	This work
MH221	MK1482, pLOW-dCas9, pVL2336-sgRNA(gfp), Ery^R, Cam^R.	This work
MM75	MH225, pVL2336-sgRNA(notarget). Ery^R, Cam^R.	This work
MH270	MH225, pVL2336-sgRNA(SAOUHSC_00685)	This work
MH264	MH225, pVL2336-sgRNA(SAOUHSC_00567)	This work
MH275	MH225, pVL2336-sgRNA(dltA). Ery^R, Cam^R.	This work
AHF59	MH225, pVL2336-sgRNA(rpsF). Ery^R, Cam^R.	This work
AHF60	MH225, pVL2336-sgRNA(vraF). Ery^R, Cam^R.	This work
AHF56	MH225, pVL2336-sgRNA(ezrA). Ery^R, Cam^R.	This work
AHF61	MH225, pVL2336-sgRNA(nrdF). Ery^R, Cam^R.	This work
MK1736	MH225, pVL2336-sgRNA(SAOUHSC_00678)	This work
MK1737	MH225, pVL2336-sgRNA(SAOUHSC_00892)	This work
AHF64	MH225, pVL2336-sgRNA(pbp4). Ery^R, Cam^R.	This work
AHF54	MH225, pVL2336-sgRNA(kapB). Ery^R, Cam^R.	This work
MK1661	MH225, pVL2336-sgRNA(ebpS). Ery^R, Cam^R.	This work
MK1662	MH225, pVL2336-sgRNA(SAOUHSC_00659). Ery^R, Cam^R.	This work
MK1663	MH225, pVL2336-sgRNA(mvaS). Ery^R, Cam^R.	This work
MK1664	MH225, pVL2336-sgRNA(sgtB). Ery^R, Cam^R.	This work
MK1667	MH225, pVL2336-sgRNA(mvaK2). Ery^R, Cam^R.	This work
MK1654	MH225, pVL2336-sgRNA(sagB). Ery^R, Cam^R.	This work
MK1655	MH225, pVL2336-sgRNA(aroB). Ery^R, Cam^R.	This work
MK1656	MH225, pVL2336-sgRNA(aroA). Ery^R, Cam^R.	This work
MK1657	MH225, pVL2336-sgRNA(aroC). Ery^R, Cam^R.	This work
MK1658	MH225, pVL2336-sgRNA(aroD). Ery^R, Cam^R.	This work
MK1659	MH225, pVL2336-sgRNA(aroK). Ery^R, Cam^R.	This work
MK1660	MH225, pVL2336-sgRNA(aroA2). Ery^R, Cam^R.	This work
MK1560	NCTC8325-4, Δ*pbp4*::spc, Spc^R	This work
MK2111	NCTC8325-4, Δ*pbp4*::spc, pLOW-P_{spac}-*pbp4*. Ery^R , Spc^R	This work
MK1718	NCTC8325-4, Δ*kapB*::spc. Spc^R	This work
MK2113	NCTC8325-4, Δ*kapB*::spc. , pLOW-P_{spac}-*pbp4*. Ery^R Spc^R	This work

Ery^R = erytromycinresistent, Cam^R = chloramphenicol resistant, Spc^R = spektinimycinresistent

Table S19. List of oligos

Oligo name	Sequence (5' – 3')
mvh54_Pspac_Rev	AGCAGAGGTTGTTACTGCTC
mvh55_intro_lacO_Pspac	GAGCAGTAACAACCTCTGCTAATTGTGAGCGCTCACAATTCTGAAAAATTTTGCAAAAAGTTGT
mk411_pbp4_up_F_NcoI	ACGTCCATGGAAATAAGACAACGAGTCATGGA
mk412_pbp4_up_R_overaad	GATCCTAGGTGGGCCCAATCCTTGTCGCTGGATTAGCAC
mk413_pbp4_down_F_overaad	CTCGAGCGGCCGCATAGTGTAGCTTGTGCATATGGTGTC
mk414_pbp4_down_R_BamHI	TCGAGGATCCAACATGATTAGTGACCCAACTA
mk188_aad9_up_F	ATTGGGCCCACCTAGGATC
mk189_aad9_down_R	ACTATGCGGCCGCTCGAG
mk619 _pbp4_RBS_F_SalI	TGCAGGTCGACGGTACCAAAGAGGAGAAAGGATCTATGAAAAATTTAATATCTATTATCATC
mk620 _pbp4_R_NotI	AGTCGCGGCCGCGCAACTTGTCCGTTTTTAGTATG
mk621 _kapB_RBS_F_SalI	TGCAGGTCGACGGTACCAAAGAGGAGAAAGGATCTATGAAGCTATATCGATTTTCACAC
mk622 _kapB_R_NotI	AGTCGCGGCCGCGTATTTAAATAAGAAGAAACACGTCA
AHF13_kapB_up_F_bsaI	TTGGCAGGTCTCCCTATGCGAAGCCCAGAATAATGTAAT
AHF14_kapB_up_R_bsaI	TTGGCAGGTCTCCGGACGTGTTTCTTCTTATTTAAATACTTCTG
AHF15_aad9_F_bsaI	TTGGCAGGTCTCGGTCCCCACCTAGGATCGAATCCC
AHF16_aad9_R_bsaI	TTGGCAGGTCTCGGCGAGGCCGCGGTAATAAAC
AHF17_kapB_dwn_F_bsaI	TTGGCAGGTCTCCTCGCCTCCCTTAAAAGTATGTTAATATATATGTATCA
AHF18_kapB_dwn_R_bsaI	TTGGCAGGTCTCCCTGCTAAAAACATAAAAAGCAACCTCAACTAT
AHF19_aad9_F_bsaI	TGTCGAGGTCTCCTGATTGGGCCCACCTAGGATCG
AHF19_aad9_R_bsaI	TGTCGAGGTCTCCTACTATGCGGCCGCTCG
OVL2127_pCG248-MutBsmBI-F	ATATCGTCTCAGGTTAATGTCATGATAATAATGGTTTCTT
OVL2128_pCG248-MutBsmBI-R	ATATCGTCTCGAACCTCACAGCTTGTCTGTAAGCGGAT
OVL2152_Read1-IF-F	TTCGGTCGACAGATCTTCGTCGGCA
OVL2153_P7-IF-R	CGGTACCCGGGGATCCCAAGCAGAAGACGGCATACGAG

Legends supplementary tables

Table S1. Sequences and off-target analysis of sgRNAs in the CRISPRi library. sgRNAs are numbered 1-1928 and the targeted locus tag is indicated along with the full 20-bp sgRNA sequences and the 12 bp core sequences. The full off-target analysis against the NCTC8325-genome (CP000253) is also shown. See de Bakker et al (6) and https://github.com/veeninglab/CRISPRi-seq for full explanation of column headings.

Table S2. sgRNA targets in the NCTC8325 genome. A list of all locus tags in the NCTC8325 genome along with the corresponding sgRNA targeting each of them. The transcriptional unit (as defined by Mäder et al. (7)), the gene name, position of the start and end of the gene (in the NCTC8325-genome), and which strand the gene is located at (1 or -1) are also shown.

Table S3. **Non-targeted locus tags in the NCTC8325 genome**. A list of the locus tags not targeted by any sgRNA. Position of the start and end of the gene (in the NCTC8325-genome) and which strand the gene is located at (1 or -1) are shown. The reason for the lack of targeting sgRNA is also indicated.

Table S4. **sgRNA targets in *S. aureus* MRSA252**. All sgRNAs in the library and their targets in *S. aureus* MRSA252. See de Bakker et al (6) for full explanation of column headings.

Table S5. **sgRNA targets in *S. aureus* RF122**. All sgRNAs in the library and their targets in *S. aureus* RF122. See de Bakker et al (6) for full explanation of column headings.

Table S6. **sgRNA targets in *S. aureus* Mu50**. All sgRNAs in the library and their targets in *S. aureus* Mu50. See de Bakker et al (6) for full explanation of column headings.

Table S7. **sgRNA targets in *S. aureus* Newman**. All sgRNAs in the library and their targets in *S. aureus* Newman. See de Bakker et al (6) for full explanation of column headings.

Table S8. **sgRNA targets in *S. aureus* JE2**. All sgRNAs in the library and their targets in *S. aureus* JE2. See de Bakker et al (6) for full explanation of column headings.

Table S9. **sgRNA targets in *S. aureus* COL**. All sgRNAs in the library and their targets in *S. aureus* COL. See de Bakker et al (6) for full explanation of column headings.

Table S10. **sgRNA targets in *S. aureus* DSM2031**. All sgRNAs in the library and their targets in *S. aureus* DSM2031. See de Bakker et al (6) for full explanation of column headings.

Table S11. Raw counts NCTC8325-4 CRISPRi-seq.

Table S12. **Gene fitness analysis of NCTC8325-4 as determined by CRISPRi-seq**. Results from the 12-generation experiment and 20-generation experiment are shown, including the log2 fold change (log2FC) between the induced and uninduced condition, the adjusted p-value (padj) and conclusion about significance ("essential" if $log_2FC<-1$, $P_{adj}<0.05$, otherwise "neutral"). Whether each sgRNA target is considered conserved essential based on previous transposon mutagenesis screens is also indicated.

Table S13. **New essential targets in NCTC8325-4**. sgRNA targets marked as essential in the CRISPRi-seq screens (Table S12), but not be reported as essential in previous transposon mutagenesis screens. The sgRNAs and their targeted genes are indicated. The possibility for the sgRNAs to affect neighbouring essential genes is also indicated.

Table S14. Raw counts Newman CRISPRi-seq.

Table S15. **Gene fitness analysis of *S. aureus* Newman by CRISPRi-seq**. Results from the 12-generation CRISPRi-seq experiment in *S. aureus* Newman (shaded in orange), including the log_2 fold change (log2FC) between the induced and uninduced condition, the adjusted p-value (padj) and conclusion about significance ("essential" or "costly" if $|log_2FC|>1$, $P_{adj}<0.05$, otherwise "neutral"). The results of the corresponding 12-generation experiment in NCTC832-4 are also included for comparison (as in Table S12).

Table S16. Raw counts NCTC8325-4 dalbavancin CRISPRi-seq.

Table S17. CRISPRi-seq screen to identify genes influencing dalbavancin susceptibility. Log_2 fold-changes between treated and non-treated samples are shown for each sgRNA as well as the corresponding p-values (padj). Significant differences indicated as follows: "essential"; genes where knockdown is expected to give increased sensitivity, "costly"; knockdown expected to give reduced sensitivity.

Table S20. **sgRNA oligos used in the library**.

References

1. Love MI, Huber W, Anders S. 2014. Moderated estimation of fold change and dispersion for RNA-seq data with DESeq2. Genome Biol 15:550.
2. Monk IR, Tree JJ, Howden BP, Stinear TP, Foster TJ. 2015. Complete bypass of restriction systems for major *Staphylococcus aureus* lineages. MBio 6:e00308.
3. Novick R. 1967. Properties of a cryptic high-frequency transducing phage in *Staphylococcus aureus*. Virology 33:155-166.
4. Duthie ES, Lorenz LL. 1952. Staphylococcal coagulase; mode of action and antigenicity. J Gen Microbiol 6:95-107.
5. Stamsås GA, Myrbråten I, Straume D, Salehian Z, Veening J-W, Håvarstein LS, Kjos M. 2018. CozEa and CozEb play overlapping and essential roles in controlling cell division in *Staphylococcus aureus*. Mol Microbiol 109:615-632.
6. de Bakker V, Liu X, Bravo AM, Veening JW. 2022. CRISPRi-seq for genome-wide fitness quantification in bacteria. Nat Protoc 17:252-281.
7. Mader U, Nicolas P, Depke M, Pane-Farre J, Debarbouille M, van der Kooi-Pol MM, Guerin C, Derozier S, Hiron A, Jarmer H, Leduc A, Michalik S, Reilman E, Schaffer M, Schmidt F, Bessieres P, Noirot P, Hecker M, Msadek T, Volker U, van Dijl JM. 2016. *Staphylococcus aureus* transcriptome architecture: from laboratory to infection-mimicking conditions. PLoS Genet 12:e1005962.
